# Exploring use of unsupervised clustering to associate signaling profiles of GPCR ligands to clinical response

**DOI:** 10.1038/s41467-019-11875-6

**Published:** 2019-09-09

**Authors:** Besma Benredjem, Jonathan Gallion, Dennis Pelletier, Paul Dallaire, Johanie Charbonneau, Darren Cawkill, Karim Nagi, Mark Gosink, Viktoryia Lukasheva, Stephen Jenkinson, Yong Ren, Christopher Somps, Brigitte Murat, Emma Van Der Westhuizen, Christian Le Gouill, Olivier Lichtarge, Anne Schmidt, Michel Bouvier, Graciela Pineyro

**Affiliations:** 1 Department of Pharmacology and Physiology, Faculty of Medicine, Université de Montréal, Montréal, QC H3T 1J4 Canada; 2 CHU Sainte-Justine research center, Montréal, QC H3T 1C5 Canada; 3 Baylor College of Medicine, Houston, TX 77030 USA; 4 Pfizer Inc, Groton, CT 06340 USA; 5 College of Medicine, Member of QU Health, Qatar University, Doha, Qatar; 6 Institute for Research in Immunology and Cancer, Department of Biochemistry and Molecular Medicine, Université de Montréal, Montréal, QC H3T 1J4 Canada; 7 Present Address: Apollo Therapeutics LLP, Stevenage Bioscience Catalyst, Gunnels Wood Road, Stevenage, SG1 2FX UK; 8 Present Address: Pfizer Inc, La Jolla, CA 92121 USA; 9 Present Address: Decibel Therapeutics, 1325 Boylston Street, Boston, MA 02215 USA; 10 Present Address: Monash Institute of Pharmaceutical Sciences, Parkville, VIC 3052 Australia

**Keywords:** Computational biology and bioinformatics, Drug screening, Pharmacology

## Abstract

Signaling diversity of G protein-coupled (GPCR) ligands provides novel opportunities to develop more effective, better-tolerated therapeutics. Taking advantage of these opportunities requires identifying which effectors should be specifically activated or avoided so as to promote desired clinical responses and avoid side effects. However, identifying signaling profiles that support desired clinical outcomes remains challenging. This study describes signaling diversity of mu opioid receptor (MOR) ligands in terms of logistic and operational parameters for ten different in vitro readouts. It then uses unsupervised clustering of curve parameters to: classify MOR ligands according to similarities in type and magnitude of response, associate resulting ligand categories with frequency of undesired events reported to the pharmacovigilance program of the Food and Drug Administration and associate signals to side effects. The ability of the classification method to associate specific in vitro signaling profiles to clinically relevant responses was corroborated using β2-adrenergic receptor ligands.

## Abstract (web summary)

Identifying ligands which activate the specific effectors driving particular in vivo drug effects remains challenging. Here, the authors apply unsupervised clustering of pharmacodynamic parameters to classify GPCR ligands into different categories with similar signaling profiles and shared frequency of report of side effects.

## Introduction

G protein-coupled receptors (GPCRs) modulate practically every aspect of human physiology and are the target of ~30% of FDA-approved medicines^1^. When activated these receptors undergo conformational changes^2,3^ that determine the type and the magnitude of signals triggered within the cell^4^. This signaling configuration supports ligand-specific activation of the different pathways^4^, and provides a theoretical opportunity for directing pharmacological stimulus toward pathways that underlie desired therapeutic responses and away from those responsible for undesired side effects^5,6^. However, in spite of this promise^5^ development of therapeutic biased ligands has yet to translate into more effective and/or better-tolerated medicines^7–11^.

Different challenges have hindered the development of clinically effective biased ligands. Except for limited examples^12–14^, we ignore the signals underlying desired and undesired clinical responses of GPCR ligands. To access this knowledge and apply it to drug discovery, it is necessary to identify signaling preferences and to associate distinct signaling profiles to desired/undesired clinical outcomes^15,16^. The way in which signaling preferences are currently identified in drug discovery efforts involves calculation of “bias factors”, an approach that uses consolidated (Log(τ/KA)) transduction coefficients to measure the extent to which a ligand preferentially activates one pathway over another^17–19^. This type of evaluation compares signals in a pairwise manner, a dichotomous approach that provides a fragmented view of a ligand’s signaling preferences across the multiplicity of pathways. Perhaps more troubling for the use of “bias factors” as descriptors of potential clinical responses is the fact that their estimated magnitudes vary with the calculation method used to produce them^15^. Finally, the same “bias factor” may describe drugs with very different efficacies at the pathways of interest^20^ further questioning the value of these measures as predictors of desired/undesired in vivo responses. In an effort to circumvent at least some of these limitations, we sought an alternative way to identify signaling preferences and classify GPCR ligands.

One of the most studied examples of how biased signaling may support development of more effective and/or better-tolerated therapeutic agents is that of opioid analgesics. Preclinical models have indicated that β-arrestin2 (βarr2) knockout mitigates constipation and respiratory depression induced by morphine^21^, pointing to the possibility that mu opioid receptor (MOR) agonists that preferentially activate G protein signaling over βarr2 recruitment could induce less of these side effects^12–14^. Here, we use this prototypical example to establish that clustering MOR ligands according to similarities in pharmacodynamic parameters for multiple responses, captures their signaling differences and preferences. We show that ligands with similar G protein/βarr responses cluster together, and provide evidence that ligands within different categories display distinct frequencies of gastrointestinal and respiratory events reported to the FDA pharmacovigilance program. Moreover, when ligands are clustered according to either G protein or βarr responses both signals directly associate to side effects. The practical value of the classification method proposed is further illustrated by the fact that ligand categories defined by similarity of G protein responses at β2-adrenergic receptor (β2AR) correlate with sympatholytic CV events and bronchoconstriction.

## Results

### Clustering ligands according to pharmacodynamic similarities

**Fig. 1:**
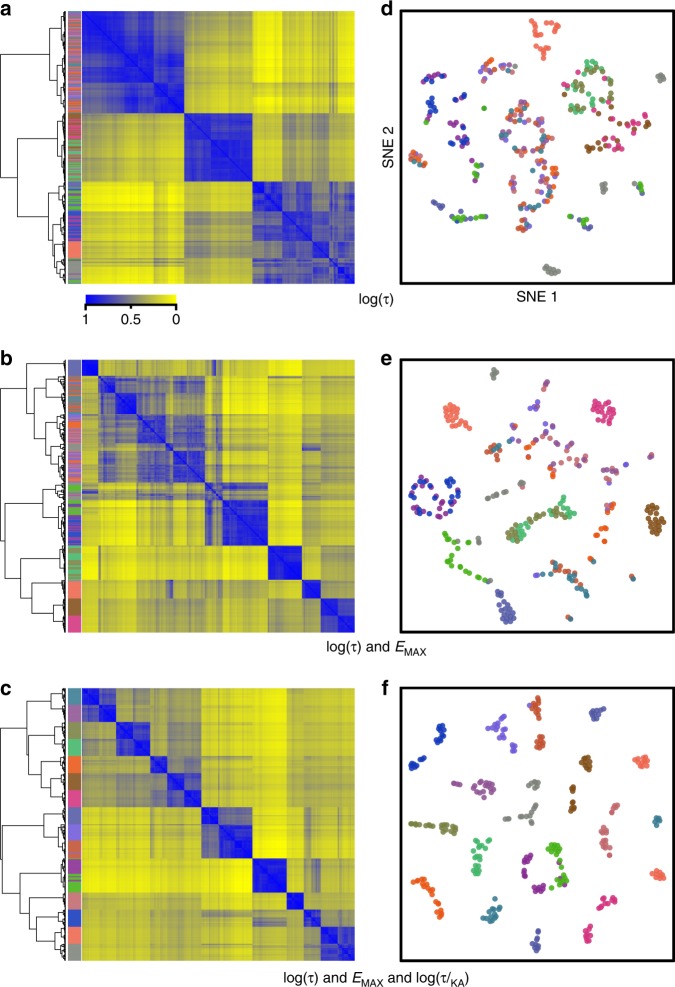
Ligands are classified according to similarities in multidimensional signaling profiles using pharmacodynamic parameters. 320 virtual compounds were defined by logistic and operational parameters to represent 16 distinct signaling profiles describing response at six different readouts. Indicated parameters were then subject to NNMF followed by k-means clustering, to produce corresponding similarity matrices that were represented as heatmaps and hierarchical clustering trees (using the R heatmap function with the metric:ward.D2) (**a**–**c**) or t-SNE plots (using the R package tsne with default parameters) (**d**–**f**). Ligands were color-coded to highlight how different combinations of parameters differentiated compounds originating from the different profiles originally defined

We sought a method to identify and group together ligands with overall similarities in a multiplicity of signaling pathways while simultaneously discerning those with overall differences in features, such as efficacy, potency, and signaling preferences. To test the ability of the method to accomplish this task independent of idiosyncrasies in experimental data sets, we generated a set of 320 virtual compounds as variations of 16 prototypical profiles characterized by a combination of pharmacodynamic features across six different readouts (see the Methods section). Profiles are shown in Supplementary Fig. 1. Criteria to classify ligands according to pharmacodynamic similarities were empirically established by generating matrices, in which each ligand was represented by individual logistic (*E*_max_, pEC50) or operational (Log(τ), pKA, Log(τ/KA)) parameters, as well as their combinations. Matrices were then subject to nonnegative matrix factorization (NNMF)^22^ to identify essential, nonredundant features, and k-means clustering was subsequently used to classify ligands according to these features^23^. Iterations were used to incorporate the error associated with each mean parameter value, ensuring its propagation throughout the clustering procedure (see the Methods and Supplementary Fig. 2). The result of this procedure was a ligand × ligand similarity matrix that quantifies how frequently any two compounds clustered together over the iterations. Final similarity matrices were submitted to hierarchical clustering to establish row and column ordering according to similarity, and visualized as heatmaps. Figure 1 shows heatmaps for the progressive associations of parameters leading to identification of Log(τ), *E*_max_, and Log(τ/KA) as a combination faithfully recreating the 16 profiles initially defined. Operational efficacy (τ) by itself was not sufficient to fully distinguish ligands with different profiles (Fig. 1a, d). Introducing measures of signaling capacity (*E*_max_) improved the classification (Fig. 1, e), but discrimination was not optimal unless values for transduction coefficient Log(τ/KA) were also included (Fig. 1c, f). Unlike Log(τ) and *E*_max_ values, transduction coefficients incorporate potency information^24,25^ and thus provide a different dimension on which ligands can be distinguished. In effect, Log(τ/KA) coefficients were correlated with logistic potency estimates (pEC50) (Supplementary Fig. 3a), so the classification afforded by Log(τ)-*E*_max_-pEC50 (Supplementary Fig. 3b, c) was quite similar to the one produced with Log(τ)-*E*_max_-Log(τ/KA). Profiles recreated by classifying ligands according to Log(τ)-*E*_max_-Log(τ/KA), are shown in Supplementary Fig. 4, revealing a minimal number of displaced compounds.

**Fig. 2:**
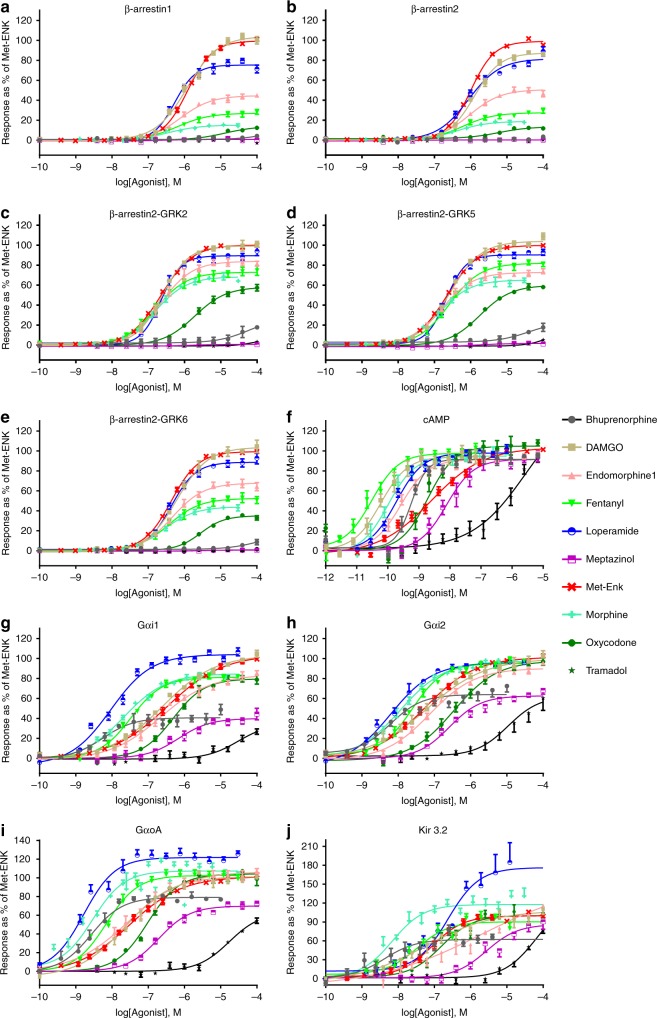
βarr recruitment and G-protein responses generated by opioid ligands. Responses for prescription opioids and known hMOR ligands were monitored using BRET-based biosensors. The results correspond to mean± SEM of at least three independent experiments, normalized to the maximal effect of Met-ENK, which was tested in all experimental runs (*n* = 16–29). Curves were fit with the operational model and the logistic equation (the results from the logistic fit are shown). **a** βarr1 recruitment, (**b**) βarr2 recruitment, (**c**) βarr2 recruitment in presence of GRK2, (**d**) βarr2 recruitment in presence of GRK5, (**e**) βarr2 recruitment in presence of GRK6, (**f**) cAMP, (**g**) Gαi1 activation, (**h**) Gαi2 activation, (**i**) GαoA activation, and (**j**) Kir3.1/3.2 activation. Net BRET values for Met-ENK are shown in Supplementary Fig. 5 and dose–response curves for all novel compounds appear in Supplementary Fig. 6. Operational and logistic parameters provided in Supplementary Data 1. Source data are provided as a source data file

**Fig. 3:**
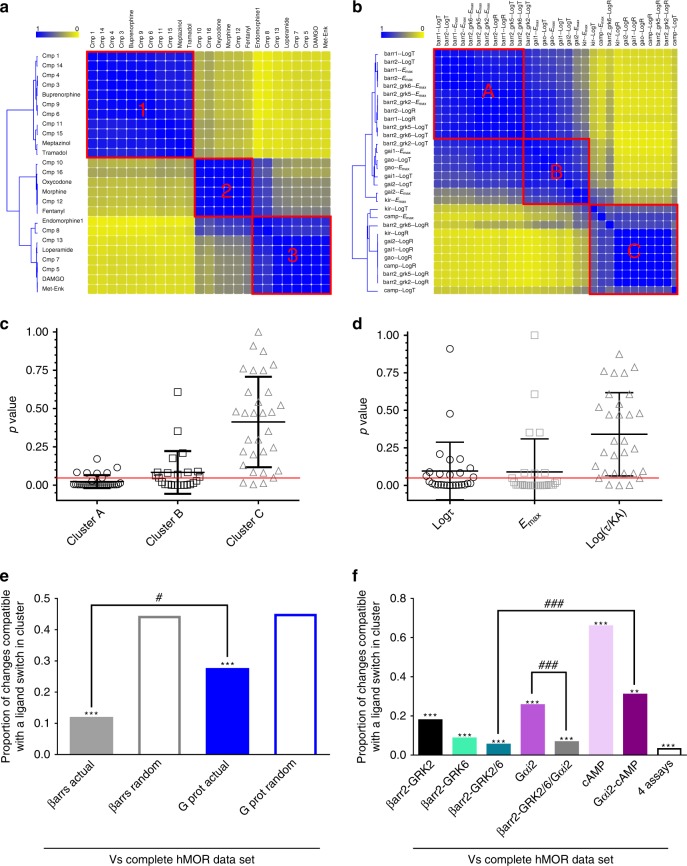
Assignment of hMOR ligands into clusters is primarily driven by βarr responses. Ligand (**a**) and parameter (**b**) similarity heatmaps for the complete hMOR data set. Yellow and blue, respectively indicate ligands/parameters that never or always cluster together. Distribution of parameters describing ligands within clusters shown in (**a**) was compared to their distribution in the whole population using a two-sample Kolmogorov–Smirnov test. Resulting *p-*values were plotted according to clusters shown in (**b**) (**c**) or to parameter type (**d**), mean ± SD are also shown. Red line: *p* = 0.05. Similarity matrices corresponding to partial data sets for βarr- or G-protein-mediated responses were compared with the complete, reference hMOR data set. Filled bars: proportion of ligands changing clusters when comparing actual βarr and G-protein data sets to the reference; empty bars: proportions observed by comparing simulations of random clustering to the reference data set. ***p < 0.001; *-z*score βarr: −5.375; *z*-score G protein: −6.092. ^#^*p* < 0.05; *z-*score difference: −2.22 (**e**). Similarity matrices generated for indicated partial responses were compared with the hMOR reference matrix. The results for actual data matrices are shown while results for random simulations were omitted. **p < 0.01; ***p < 0.001 comparing partial data sets to their randomized controls; *z-*scores for comparisons between actual and randomized data: βarr2-GRK2: −12.724; βarr2-GRK6: −10.583; βarr2-GRK2/GRK6:− 8.835; Gαi2: −7.315; cAMP: 6.297; Gαi2- cAMP: −2.351; four assays −8.541; βarr2-GKR2/6/Gαi2: −7.391. ^###^*p* < 0.001; *z*-score difference: βarr2-GRK2/6 vs. Gαi2/cAMP: −3.308; *z-*score difference: Gαi2 vs βarr2-GRK2/6-Gαi2: 3.754 (**f**). Source data provided in Supplementary Data 1 and as a source data file

We then applied the proposed classification strategy on experimental data. Multidimensional signaling profiles for this analysis were generated using ten different BRET-based biosensors that monitor βarr recruitment and G protein signaling. G-protein signaling was monitored through conformational rearrangements within Gα_i1-2_/_oA_β_1_γ_2_ heterotrimers^26^, at the interface of Gβ_1_γ_2_/Kir3 channel subunits^27^, or as changes in cAMP levels^28,29^. βarr recruitment to the receptor was assessed for βarr1, βarr2, and βarr2 in presence of GRK2, GRK5, or GRK6 to account for possible impact of expression differences between the screening system (HEK 293) and target neurons where GRK levels are higher^30^. Net BRET values obtained in cells co-expressing human MORs (hMOR) and different biosensors in presence/absence of the endogenous ligand Met-Enkephalin (Met-ENK) are shown for reference in Supplementary Fig. 5. Concentration response curves (CRCs) for 10 known opioids (Fig. 2), and 15 novel compounds (Supplementary Fig. 6) identified in the context of a screening campaign at Pfizer Inc.^31^, were then generated and analyzed with the logistic equation and the operational^32^ model. Each ligand was phenotypically described by corresponding τ, *E*_MAX_, and Log(τ/KA) values (± SEM) derived from 5 G protein- and 5 βarr-related responses (Supplementary Data 1). These were analyzed with NNMF/k-means clustering as above and represented as heat maps for ligands (Fig. 3a) and for parameters (Fig. 3b).

### Ligands within the same cluster share quality and magnitude of response

An essential pharmacological question is to identify the pathways and pharmacodynamic properties primarily responsible for ligand clustering. The overall resemblance among relative magnitudes of operational and logistic parameters from different functional readouts is shown in Fig. 3b delineating three clusters of parameters. To further characterize differences among ligand categories, we investigated whether the magnitude of parameters describing ligand response in each assay was different across the three clusters of ligands. To do so, we used a Kolmogorov–Smirnoff test to compare the distribution of parameter values in each cluster to that of the overall population (detailed in Supplementary Fig. 7). Only certain parameters in each cluster contributed to ligand discrimination, and they did so to different extents (Fig. 3c). Those in cluster A had the most weight, as 29.9% of comparisons identified at least one distribution of parameters significantly different from that of the whole population. Overall, 14.9% of comparisons in cluster B and 3.5% in cluster C also significantly contributed to ligand discrimination. Alternatively, ordering parameters by type (Fig. 3d) revealed that efficacy-related parameters (*E*_max_ and τ) had the most weight in the classification (52.0% and 36.0% of the comparisons, respectively, identified distributions different from the whole population) while Log(τ/KA) played a smaller role separating primary compound clusters (11.0%). Finally, Supplementary Table 1 shows assay parameters significantly contributing to ligand clustering. The diversity of signals determining cluster assignment distinguishes this multidimensional classification from dichotomous comparisons underlying bias magnitudes.

**Fig. 4:**
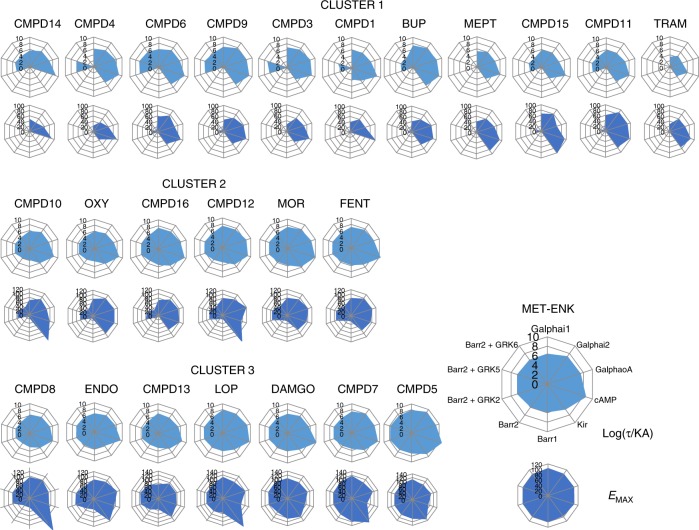
Graphic representation of operational and logistic parameters for hMOR ligands populating different clusters. Operational transduction coefficients (Log(τ/KA)) and logistic *E*_max_ values derived from concentration response curves generated by hMORs at ten different biosensors were represented as radial graphs. Each radius corresponds to the magnitude of Log(τ/KA) or *E*_max_ values. Transduction coefficients are in logarithmic scale, *E*_max_ values were normalized to maximal Met-ENK response, and are presented on linear scale. Source data provided in Supplementary Data 1

Importantly, despite independence from bias magnitudes, the proposed classification strategy still allows to evaluate relative contributions of βarr and G protein signaling to ligand assignment to clusters. To access this information, drugs were re-clustered using subsets of the data corresponding exclusively to G protein (Supplementary Fig. 8a) or to βarr (Supplementary Fig. 8b) assays, and the resulting similarity matrix produced with each partial data set was compared with the matrix generated using the complete set of values. Differences between matrices were quantified as described in the Methods section and Supplementary Fig. 9, and expressed as the proportion of changes in ligand distances that were compatible with a switch in clusters between the two compared matrices. Clusters generated with βarr data differed by only 11.5% from clusters produced with the complete data set (Fig. 3e), underscoring the similarity of drug classes defined by βarr signaling patterns and complete signaling profiles. The partial data set for G protein responses differed by 27.2% (Fig. 3e) from the complete ligand similarity matrix. Thus, although clusters generated with βarr or with G protein data sets resembled clusters produced with the complete data set more than did clusters generated with the corresponding randomized values, initial ligand clustering was more faithfully recreated by βarr responses (Fig. 3e), indicating that that this signal was the one primarily driving classification in the complete matrix. Profiles graphically representing *E*_max_ and Log(τ/KA) values for G protein and βarr readouts further highlight how the analysis clustered ligands according to type and magnitude of responses elicited (Fig. 4). Ligands in cluster #3 were full, reasonably balanced agonists characterized by maximal effects at βarr and G protein readouts. Ligands in cluster #2 were partial agonists for G protein-mediated responses with measurable βarr recruitment only in presence of overexpressed GRKs, while ligands in cluster #1 displayed minimal or no βarr recruitment and G protein responses were overall smaller than in cluster #2.

While it may be feasible to monitor ten different signaling outcomes for a small group of ligands, it is unlikely that this could be done in high-throughput screening or structure–activity profiling. Hence, once we had identified the signals that contributed the most to drug classification, we determined whether a reduced number of assays could convey similar diversity. To this end, βarr2 + GRK2, βarr2 + GRK6, Gαi2, and cAMP were chosen as respective prototypes of βarr- and G protein responses. Similarity matrices generated from these individual signals or from their combinations were compared with the complete similarity matrix. As above, we compared each partial data set to the complete set of hMOR parameters, and then established if the proportion of ligands switching clusters was less than that observed for the corresponding randomized data set. For cAMP, the proportion of changes were larger than the expected random value (Fig. 3f), indicating minimal contribution of this signal to ligand classification. In contrast, for clusters generated with the other data subsets, the proportion of ligands switching clusters was significantly smaller than the random expectation (Fig. 3f), indicating that each of these signals significantly supported ligand discrimination in the complete data set, albeit to different extents.

The combination of βarr2 + GRK2 and βarr2 + GRK6 data was the best at reproducing clustering obtained with the complete hMOR similarity matrix (91.4%; Fig. 3f). In comparison, the Gαi2 data set either combined with cAMP or in isolation moderately recreated the clusters of the complete matrix (Gαi2 = 74.4%, Gαi2 + cAMP = 69.0% differences). Combining all four assays added little extra precision as compared with βarr2 + GRK2 with βarr2 + GRK6 (Fig. 3f). Thus, taken together, these data indicate that it is possible to first identify the signals that primarily contribute to signaling diversity of a group of compounds at a given receptor, and then use these signals as surrogate readout for screening campaigns over large collections of compounds.

### Ligand clusters are informative of possible side effects

Preclinical studies suggest that signaling diversity of MOR agonists provides a means of improving tolerability of opioid analgesics^12–14^. Therefore, it was of interest to determine if the pharmacodynamic clusters just defined could inform us about clinical side effects of ligands in each category. To address this issue, we first used standardized gamma (SD gamma) scores^33^ to identify adverse events most frequently reported for opioids in the Food and Drug Administration’s pharmacovigilance data base (Adverse Effects Report System (AERS)), and then calculated the scores of these events for each of the prescription opioids used in the study. These measures of side effect prevalence were associated to ligands in the different clusters by using the Euclidian distance between ligands in the similarity matrix. Tramadol was set as the arbitrary origin, and distances separating the rest of prescription opioids from tramadol in the Log(τ)-*E*_max_-Log(τ/KA) matrix were consigned as measures of ligand similarity. These measures were then correlated to the SD gamma scores for each ligand’s side effects. A complete list of the 80 events considered along with *r*^2^ and *p-*values for each correlation is provided in Supplementary Data 2. Correlations that were significant (*p* ≤ 0.05) and/or explained at least 60% of the variance (*r*^2^ ≥ 0.60) were considered. Applying these criteria, ligand position in the Log(τ)-*E*_max_-Log(τ/KA) matrix was correlated to 6 out of a total of 80 associations considered (7.5%), including gastrointestinal (GI) events, respiratory depression, and somnolence (Table 1), all typically associated to opioid therapy^34,35^. These correlations confirm that signaling categories defined by unsupervised clustering can be associated to distinct frequency of report of undesired effects of opioid ligands.

**Table 1:** Pharmacodynamic and structural categories associate with frequency of report of undesired events for clinically available hMOR ligands^#^

Undesired events associated with	Type of side effect	Preferred term	R square	*p-*value
Functional categories defined by Log(τ)–*E*_max_–Log(τ/KA)	Gastrointestinal/nutrition	Cachexia	0.66	0.05
		Faecaloma	0.86	0.01
		Gastrointestinal motility disorder	0.81	0.01
	Respiratory events	Respiratory depression	0.62	0.11
		Respiratory rate decreased	0.60	0.13
	Sleep disorder	Somnolence	0.60	0.12
Functional categories defined by Log(τ)-*E*_max_	Gastrointestinal/nutrition	Cachexia	0.66	0.05
		Faecaloma	0.92	0.00
		Gastrointestinal motility disorder	0.62	0.06
	Respiratory events	Hypoventilation	0.68	0.08
		Oxygen saturation decreased	0.61	0.12
		Respiratory depression	0.78	0.05
		Respiratory rate decreased	0.69	0.08
	Sleep disorder	Somnolence	0.75	0.06
Structural categories	Respiratory events	Hypopnoea	0.65	0.10
		Yawning	0.79	0.04
	Neuropsychiatric	Withdrawal syndrome	0.74	0.06
	Change in drug response	Analgesic drug level increased	0.70	0.08
		Drug effect decreased	0.64	0.10
		Drug effect increased	0.90	0.01
		Therapeutic response increased	0.89	0.02
		Therapeutic response decreased	0.74	0.06
	Pain	Breakthrough pain	0.69	0.08
		Complex regional pain syndrome	0.64	0.11
	Itching	Pruritus generalized	0.86	0.02
	Others	Therapy cessation	0.76	0.05

^#^Only significant correlations, and correlations that explained 60% or more of the variance were considered; full information in Supplementary Data 2. Similarity and SD gamma scores for Buprenorphine, Fentanyl, Morphine, Oxycodone, and Tramadol were used to establish correlations. Loperamide was additionally included in correlations for gastrointestinal reports. Source data provided in Supplementary Data 2 and source data files

Log(τ) and *E*_max_ were the main determinants of ligand position in the matrix constituting 89.0% of parameters effectively grouping ligands into clusters (Fig. 3d). Not surprisingly, if ligands were classified exclusively using these efficacy-related parameters, all side effects previously associated with ligand position in the Log(τ)-*E*_max_-Log(τ/KA) matrix remained correlated with their positions in this efficacy-only matrix (Table 1). Actually, categories driven by efficacy measures associated with more side effects than clusters established by including Log(τ/KA) as an additional classification criterion (Table 1). Thus, even if functional affinity information within transduction coefficient affords better discrimination of ligands, it also acts as a confounder for cluster association to side effects.

### Associating side effects to specific signals

Preclinical studies have suggested that MOR agonists that preferentially engage G protein over βarr responses could display less gastrointestinal and respiratory side effects in the clinic^12–14^. Hence, we were interested to find out whether AERS reports for opioids would distinctively correlate with ligand categories defined either by G protein or βarr signaling. There has been considerable debate as to whether biased signaling is best identified using Log(τ/KA) transduction coefficients^17,24,25^ or efficacy-related measures^36,37^. Hence, partial matrices in which drugs were classified according to G protein or βarr responses were generated using either Log(τ)-*E*_max_ or Log(τ)-*E*_max_-Log(τ/KA) as classification criteria. Supplementary Fig. 10 shows how frequency of faecaloma report correlates to similarity scores in these four partial matrices. Considering the classification based exclusively on Log(τ)-*E*_max_ values, categories defined by βarr and G protein responses were both correlated to faecaloma report (Supplementary Fig. 10a), implying no differential association of these signals to the undesired event. In contrast, when Log(τ/KA) coefficients were additionally considered, faecaloma report correlated to βarr, but not G protein responses (Supplementary Fig. 10b). However, it is worth considering how inclusion of Log(τ/KA) values breaks the correlation previously established with efficacy-based categories. BUP has a high transduction coefficient that cannot be distinguished from those of fentanyl (FEN) or loperamide (LOP), causing the partial agonist to move closer to these efficacious ligands in the matrix. Yet BUP’s transduction coefficient is driven by its high affinity^38,39^, and regardless of its position among efficacious agonists its side effects profile remains determined by its partial efficacy, disrupting the correlation.

Opioid modulation of acute ileum contractility is G protein-driven by effectors that hyperpolarize myenteric neurons and inhibit neurotransmitter release by vagal terminals^40–42^^,^. We used this G protein-mediated response to ascertain that failure to correlate faecaloma report to categories partly defined by Log(τ/KA) was not due to the method itself. In effect, as shown in Supplementary Fig. 10c–e, frequency of faecaloma report correlated with Log(τ) but not Log(τ/KA) values describing ligand inhibition of ileum contraction.

### Signaling and structural clusters convey complementary information about side effects

Structural criteria are used to classify, compare, and infer possible commonalities of in vivo responses for drug candidates^43^. We therefore compared categories of ligands defined by pharmacodynamic and structural criteria. Ligand structure was described using Tanimoto values^44^ derived from standard fingerprint representations (Supplementary Data 3–5), and these values were then clustered using the same NNMF/k-means method as previously applied on signaling profiles. The resulting clusters are shown in Supplementary Fig. 11a, and representatives of each structural group are provided in Supplementary Fig. 12. Structural and pharmacodynamic similarity matrices were then compared, indicating that 36.0% of changes in ligand distance were compatible with a switch in cluster when the two different criteria were applied. This value was significantly lower when compared with 43.5% switches observed using randomized structural data (*z*-score = −2.803; *p* < 0.01), denoting some degree of statistical similarity between signaling and structural categories (Source data provided). However, the degree of similarity was low as schematically represented in Supplementary Fig. 11b. In keeping with this notion, clusters based on chemical structures were correlated with a different set of reported events than those associated with the pharmacodynamic clusters (Table 1). In particular, ligand distances in the matrix generated with chemical structures correlated with 12.5% of reported events, including pruritus, a typical opioid associated complaint^45^, as well as with reports of withdrawal and fluctuations in response and drug levels (Supplementary Data 6). Since structure determines pharmacokinetic properties, it is not surprising that structural categories associate with fluctuations in drug effects and even withdrawal symptoms^46^. On the other hand, and in spite of pharmacodynamic properties also being determined by structure, categories based on structural fingerprint representations failed to identify any of the events that associated with signaling categories, emphasizing the value of complementing structural information with a signal-based classification.

### Ligand clusters generated with different GPCRs

**Fig. 5:**
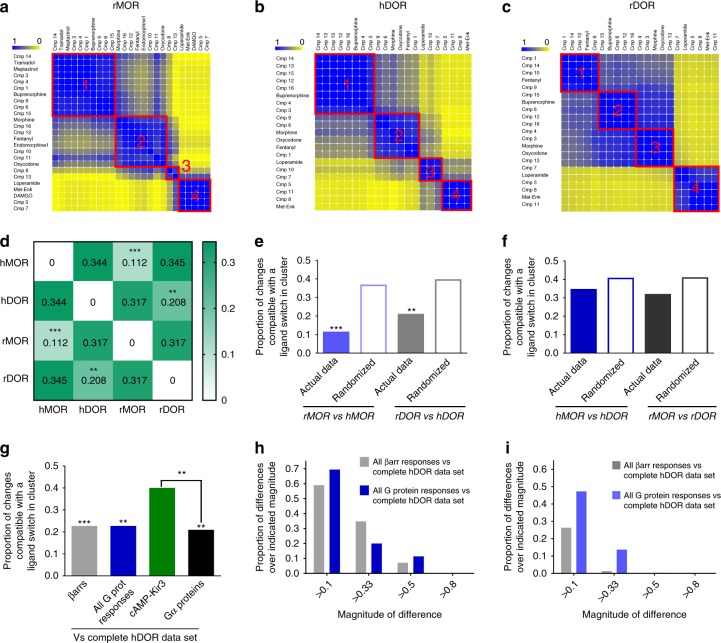
Signaling profiles of opioid receptor ligands are conserved across species but not receptor subtypes. Ligand similarity heatmaps for rat MOR (**a**), human DOR (**b**), and rat DOR (**c**) data sets. Yellow and blue, respectively, indicate ligands/parameters that never or always cluster together. Proportion of ligands changing cluster for indicated comparisons; **p < 0.01, ***p < 0.001 comparing actual to randomized data sets as in Fig. 3 (**d**). Similarity matrices for the same receptor compared across species. Filled and empty bars: proportion of ligands changing clusters when, respectively, comparing actual data and random clustering simulations to the reference matrix; **p < 0.01, ***p < 0.001; *z*-score MOR subtypes: −7.153; *z*-score DOR subtypes: −2.742 (**e**). Similarity matrices for receptor subtypes within species compared as in (**e**). Actual and randomized data sets did not differ: *z*-score hMOR vs. hDOR: −1.502; *z*-score rMOR vs. rDOR subtypes: −1.567 (**f**). Similarity matrices for βarr- or G protein-partial data sets were compared with the complete hDOR matrix as in (**e**); only comparisons for actual data are shown; **p < 0.01, ***p < 0.001; *z*-scores versus corresponding randomized data: βarr: −3.309; G protein: −2.644; cAMP-Kir3.2: −0.309, Gα proteins: −3.286. ^##^*p* < 0.01; *z-*score difference: cAMP-Kir3.2 vs. Gα proteins: 2.515. *p* = 0.256; *z*-score difference βarrs vs. All G prot responses: −0.656 (**g**). A difference was calculated between every value_ij_ in the complete hDOR matrix and corresponding value_ij_ in indicated partial data sets. Histogram shows the fraction of differences with absolute value above indicated thresholds (**h**). Calculations described in (**h**) were repeated for the complete hMOR matrix and partial data sets for βarr or G protein (**i**). Source data provided in Supplementary Data 7, 8, and 9 and source data files

We next examined whether clustering analysis could reveal pharmacodynamic similarities and differences among ligand responses generated at different opioid receptor subtypes. To do so, we used the same set of biosensors as for hMORs to monitor ligand activity at rat MORs (rMORs), human delta opioid receptor (hDORs) and rat DORs (rDORs). Corresponding input matrices containing logistic and operational parameters for each receptor (Supplementary Data 7–9) were analyzed as before to yield individual similarity matrices and associated heatmaps (Fig. 5a–c). Differences in clustering across receptor subtypes and species were evaluated by comparing similarity matrices for each receptor and are summarized in Fig. 5d. These comparisons revealed that the pattern of signaling diversity of this group of opioid ligands was reasonably conserved within the same receptor from different species. Indeed, in comparisons between rat and human MORs or rat and human DORs, the proportion of changes in ligand distances that were compatible with a switch in cluster was significantly less for actual as compared with randomized data sets (Fig. 5e), confirming congruent patterns across species.

In contrast, when clusters generated with data sets from MORs and DORs within the same species were compared, their differences were statistically indistinguishable from those obtained by comparing corresponding sets of randomized data (Fig. 5f), indicating that the analysis discerned the distinct pharmacological profiles of the two receptor subtypes. To identify the source of these differences, we compared the relative contribution of βarr and G protein responses in driving ligand clustering this time according to hDOR responses. Clusters generated with each partial data set bore statistical similarity to clustering done using the complete data set, and no statistical difference was revealed between clusters produced with βarr and G protein parameters (Fig. 5g). To more precisely establish the weight of βarr and Gprotein responses to clustering of ligands according to hDOR signaling, we investigated how every value in the similarity matrix changed when considering hDOR clusters produced with the complete data set, and clusters generated with pathway-specific data sets. As shown in Fig. 5h, the variations between the complete and the G protein similarity matrices paralleled the differences between the complete and the βarr similarity matrices, indicating the two types of signals similarly contributed to the classification of ligands according to responses generated at hDORs. In contrast, and consistent with the fact that βarr recruitment was the main determinant in hMOR clustering, the differences between G protein and complete matrices were more frequent and larger than those for the corresponding βarr comparison (Fig. 5i). A graphical representation of hDOR clusters is given in Supplementary Fig. 13.

**Fig. 6:**
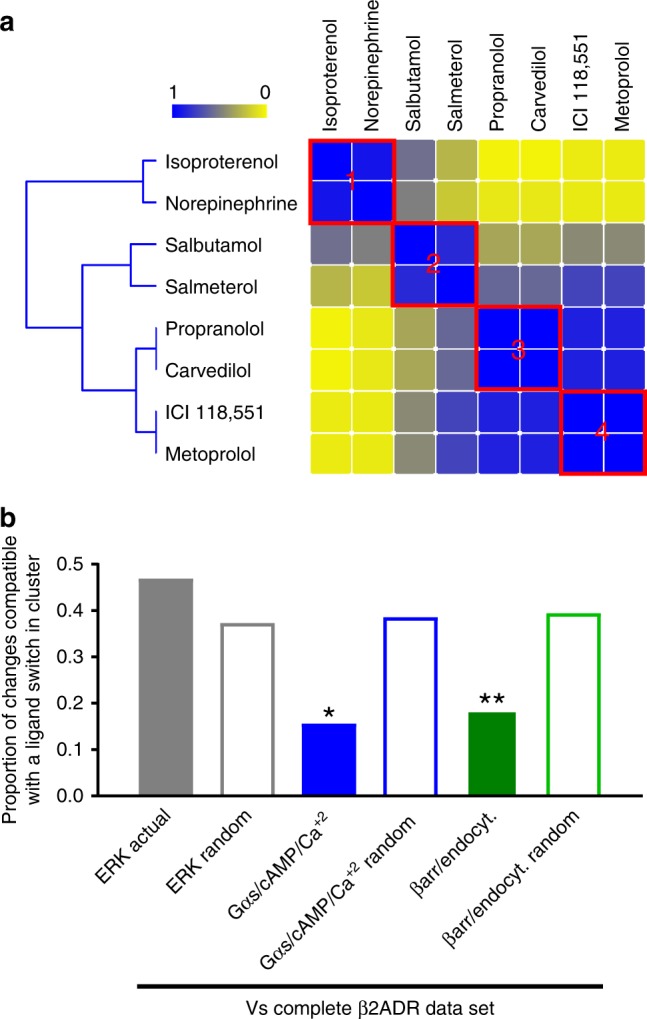
β2_ADR_ ligands cluster according to similarity in G protein and βarr-mediated responses. Ligand similarity heatmaps for hβ2_ADRs._ Yellow and blue, respectively, indicate ligands/parameters that never or always cluster together. (**a**). Similarity matrices for partial data sets corresponding to ERK, G protein- (Gαs, cAMP, Ca^+2^), and βarr2- (recruitment, endocytosis) mediated responses were compared with the reference hβ2_ADRs_ data set. Filled bars: proportion of ligands changing clusters when comparing actual ERK, G protein, and βarr2 data sets to the reference; empty bars: proportions observed comparing the reference data set to corresponding simulations of random clustering for partial matrices. *p < 0.05, **p < 0.01; *z*-score ERK: 1.523; *z*-score G protein: −2.446 and z-score βarr: −2.636 (**b**). Source data provided in Supplementary Data 10 and source data files

Finally, we assessed whether clustering according to signaling profiles could be extended to GPCRs that couple to effectors different than those activated by opioid receptors. For this purpose, we considered published^47^ and novel data generated with β2-adrenergic receptor (β2AR) ligands including: (a) G protein-dependent responses (Gαs activation, cAMP production, Ca^2+^ mobilization)^48^, (b) βarr-mediated responses (βarr2 recruitment and receptor internalization), and (c) ERK signaling, a multifaceted response involving both G proteins and βarrs^49^. Parameters describing concentration response curves for each of the readouts (Supplementary Data 10) were analyzed by NNMF and k-means to reveal four different drug categories (Fig. 6a). Cluster #1, including isoproterenol (ISO) and norepinephrine (NE), was characterized by measurable agonist efficacy at all readouts. Salbutamol (SALB) and salmeterol (SALM) in cluster #2 could be distinguished from the first category because of their minimal responses at βarr-dependent readouts. Carvedilol (CARV) and propranolol (PRO) behaved as agonists only in the ERK pathway (Cluster #3), while ICI118,555 and metoprolol (MET) had no efficacy except for inverse agonism at Gαs and cAMP assays (cluster #4). The complete signaling profiles for ligands in different clusters are provided in Supplementary Fig. 14.

As shown in Fig. 6b, partial data sets for Gαs/cAMP/Ca^2+^ and for βarr-recruitment/endocytosis recreated original clusters better than their corresponding randomized controls, indicating significant contribution of these signals to ligand clustering. To establish whether these categories were also relevant to human pharmacology, we evaluated their association to pharmacovigilance data. For this purpose, undesired cardiovascular and respiratory events most frequently reported for β2AR agonists and antagonists were first identified, and SD gamma scores representing the frequency with which these events were reported for the prescription ligands used in the study was correlated to their signaling similarity (measured as Euclidian distances in the full matrix) (Supplementary Data 11). We found that increasing distance from the agonist ISO was significantly correlated (*p* < 0.05) with increasing frequency of reports for hypotension, decrease in blood pressure, sinus bradycardia, atrioventricular block, sinus arrest, and need for inhalation therapy (Table 2). Interestingly, the first four events in this list typically correspond to reduced sympathetic tone on cardiovascular function^50^. Hence, their more frequent association with ligands that clustered furthest apart from ISO is entirely consistent with gradual loss of efficacy at β2AR. Moreover, these four events were also negatively correlated with ligand efficacy to induce Gαs, cAMP, and Ca^2+^ signaling (Table 2), an overlap that is consistent both with the well-document role of these signals in maintaining heart chrono-, and inotropism^51^ and with the fact that G protein-dependent signals significantly drove ligand clustering. Gαs, cAMP, and Ca^2+^ signaling categories also showed increasing reports of deleterious effects on respiratory function, some of them such as asthma, asthmatic crisis, status asthmaticus consistent with bronchoconstriction, as distance from ISO and β2AR antagonism increased. Thus, pharmacodynamic categories defined by as few as eight ligands were sufficiently robust to reveal a well-known association between sympatholytic CV events or manifestations of bronchoconstriction and modulation of G protein activity by β2AR ligands. The observation reinforces the notion that unsupervised clustering of multidimensional signaling profiles allows the association of signals generated in simple cellular models to possible clinical effects of GPCR ligands.

**Table 2:** Pharmacodynamic categories associate with frequency of report of undesired cardiovascular and respiratory events for clinically available β2_ADR_ ligands^#^

Correlations between SD gamma scores for prescription β2ADR ligands* and	Type of side effect	Preferred term	R square	*p-*value
Functional categories defined by Log(τ)-E_max_-Log(τ/KA)	CV	Hypotension	0.60	0.04
		Sinus bradycardia	0.58	0.05
		Blood pressure decreased	0.64	0.03
		Atrioventricular block	0.62	0.04
		Sinus arrest	0.68	0.02
	Respiratory	Inhalation therapy	0.60	0.04
Functional categories defined by Gs/cAMP/Ca2 + (partial matrix)	CV	Hypotension	0.80	0.01
		Sinus bradycardia	0.66	0.03
		Blood pressure decreased	0.81	0.01
		Atrioventricular block	0.72	0.02
		Blood pressure systolic decreased	0.64	0.03
		Sinus arrest	0.82	0.00
		Ejection fraction decreased	0.82	0.01
		Heart rate decreased	0.67	0.05
	Respiratory	Asthma	0.64	0.03
		Inhalation therapy	0.59	0.05
		Asthmatic crisis	0.74	0.01
		Status asthmaticus	0.89	0.00
		Throat irritation	0.69	0.02
		Choking	0.64	0.03

^#^Only significant correlations were considered, full information in Supplementary Data 11. Similarity and SD gamma scores for Isoproterenol, norepinephrine, salbutamol, salmeterol, propranolol, carvedilol, and metroprolol were used to establish correlations. Source data provided in Supplementary Data 11 and source data files

## Discussion

This study introduced a stepwise analysis in which GPCR ligands were organized into pharmacodynamic categories that could be then associated with clinically relevant responses. Pharmacodynamic parameters that best supported classification of ligands ((Log(τ)-*E*_max_-Log(τ/KA)) were empirically chosen by consecutively applying NNMF and k-means clustering to different combination of parameters informative of efficacies and functional affinities. The procedure was successfully applied to classify groups of ligands ranging from 8–320 in number, and representative of a multiplicity of signaling profiles.

Ligand categories generated with (Log(τ)-*E*_max_-Log(τ/KA)) values from hMOR data were primarily driven by ligand diversity in βarr signaling efficacy, but G protein responses also contributed to the classification. Measured similarity among signaling profiles of prescription opioids present in the different categories was correlated with their corresponding frequencies of AERS reports for typical opioid side effects, indicating that the categories established by applying this exploratory method may allow to establish meaningful associations between in vitro signals and clinically relevant drug actions. This notion was further supported by observations that pharmacodynamic categories defined for β2AR ligands were essentially driven by G protein responses and associated to G protein-driven sympatholytic effects^52,53^. Hence, by unveiling these well-documented associations, we established that clustering analysis of concentration–response parameters allows to associate multidimensional in vitro signaling profiles to clinical responses. Such use of curve parameters should prove beneficial for identifying signals that support specific responses of interest and for which mechanistic information is unavailable. Pharmacodynamic categories defined by efficacy-related parameters (Log(τ)-*E*_max_) had stronger and more frequent correlations to side effects than those defined by additional inclusion of affinity information provided by transduction coefficients. On the other hand, ligand differentiation was optimal when transduction coefficients were taken into account, calling for a discretionary decision on which parameters to use depending on the goal of the classification.

By classifying opioid ligands according to pathway-specific responses, it was possible to explore whether specific signals were driving typical side effects of opioids. We found that association of faecaloma report to categories defined by G protein responses was influenced by the parameters used in ligand classification. In Log(τ)-*E*_max_ matrices, G protein and βarr categories were both directly correlated with reports for this side effect, implying that weaker agonists were associated with lower frequency of reports. In contrast, the correlation with G protein responses was disrupted if Log(τ/KA) values were also considered, suggesting a scenario were G protein signaling would not associate to side effects. This divergence as compared with Log(τ)-*E*_max_ matrices is linked to the fact that despite its partial efficacy and a side effects profile consistent with partial agonism, Log(τ/KA) coefficients could not distinguish this low-efficacy–high-affinity ligand from much more efficacious agonists, such as morphine or oxycodone. In contrast to G proteins, βarr categories correlated to faecaloma report independent of the parameters used for classification, as transduction coefficients for βarr responses were consistent with BUP’s low efficacy. Log(τ/KA) transduction coefficients are largely used to identify biased ligands^17,25^, so a word of caution is warranted for bias measures driven by functional affinity since only efficacy parameters are predictive of the magnitude of in vivo responses^54^.

It is also of interest that maximal responses for βarr and G proteins decreased in parallel across the different clusters, albeit not to the same extent. Indeed, βarr signals gradually disappeared while those of G proteins grew progressively smaller without completely vanishing. Such systematic imbalance between the two types of signals has been previously reported^55^, and is akin to system bias^25^ where βarr responses are less well coupled to the receptor than G protein signals. Within this context, absence of βarr responses may simply indicate partial agonism and not signaling bias. As a matter of fact, all novel biased hMOR ligands presented herein as well as those published to date (i.e.: TRV130^12^; PZM21^13^ and Scripps compounds^14^) are partial agonists at G protein responses. This raises the possibility that currently available biased ligands could simply produce less side effects because they are partially effective at stimulating the receptor, and not necessarily because of greater efficacy for activating the G protein over βarr. A miss-interpretation of bias might be a problem for future clinical applications since a partial agonist may also produce a submaximal analgesic response. In this sense, it is of interest that βarr-G protein signaling profiles of the latest hMOR ligands^14^ resemble those obtained in this study for BUP, a partially effective analgesic^56^. It is also worth considering that the clinical profile of TRV130, a partial hMOR agonist which was clinically tested as the first biased agonist for MORs, did not significantly differ from morphine’s profile at doses with equivalent analgesic effects^10^. Finally, when PZM21 was independently tested after its initial description as biased agonist, it was shown to behave as a partial agonist in βarr and G protein readouts, and to produce respiratory depression commensurate with partial signaling^10^.

Structural similarity is another means for inferring common in vivo responses of therapeutic drug candidates early in the discovery process^43^. Here, when clusters established on the bases of signaling and structural resemblance were compared, they displayed nonrandom but marginal similarity. Different reasons could explain the low degree of similarity between categories established with structural and pharmacodynamic criteria, including incomplete representation of structural diversity of opioid ligands within the sample used, or different discriminatory power of signaling profiles and current descriptors of structural properties. Consistent with their low degree of similarity, structural, and pharmacodynamic categories were associated with different types of undesired events. Indeed, structural similarities were more frequently associated with fluctuations in therapeutic response, which are typically associated with pharmacokinetic properties^42^. On the other hand, signaling categories specifically correlated with on-target side effects, pointing to the complementarity of both approaches when characterizing a limited number of compounds of interest.

In conclusion, we presented an unsupervised classification method that incorporates distinct and complementary data sources to comprehensively describe signaling diversity of GPCR ligands. The procedure identifies signaling imbalance independent of whether bias in the response co-varies with efficacy, it was applied to a large diversity of signaling profiles and distinguishes subtle differences in signaling preferences.

## Methods

### Materials and reagents

Standard opioids were purchased from Cedarlane (Burlington, Canada) and Sigma-Aldrich (St. Louis, MO, USA). Fifteen novel compounds were provided by Pfizer Inc. (Worldwide Research and Development). (−)-Isoproterenol hydrochloride, (−)-norepinephrine, DL-propranolol hydrochloride, ( ± ) metoprolol ( + )-tartrate salt, carvedilol, and salmeterol xinafoate were purchased from Sigma-Aldrich (St Louis, MO). ICI 118,551 and salbutamol hemisulfate were purchased from Tocris Bioscience (Ellisville, MO). Coelenterazine 400a was purchased from Biotium.

### Plasmids and DNA constructs

A cleavable signal sequence of influenza hemagglutinin (MKTIIALSYIFCLVFA) and a Flag tag (MDYKDDDDA) were added to the human MOR1, rat MOR1, human DOR, and rat DOR and, their coding sequence optimized and synthetized as Strings DNA Fragments at GeneART (ThermoFisher Scientific). The DNA Strings were subcloned by Gibson assembly (New England Biolabs Canada) in pLVX-IRES-Puro (Clontech Laboratories, Inc). Untagged versions of the receptors were made by an internal NcoI deletion, removing the coding sequence of the Flag tag. Constructs encoding for GFP10-tagged receptors were made by PCR overlap; the coding sequence of each signal-peptide Flag-receptors was PCR-amplified to remove the stop codon and assembled by PCR overlap with the coding sequence of GFP10. The resulting PCR products were subcloned by Gibson assembly in pLVX-IRES-Puro. Constructs encoding the Epac-based cAMP sensor (GFP10-Epac-RlucII), RlucII-tagged Gα (αi1, αi2, αoA, αs), GFP10-Gγ1, GFP10-Gγ2, β-arrestin1-RlucII, β-arrestin2-RlucII, RlucII-Gγ2, human β2-adrenergic receptor (hβ2AR), and cmyc-hβ2AR-GFP10 were previously described (PMID: 15782186, PMID: 22534132, PMID: 23175530, PMID: 24309376, PMID: 19584306, PMID: 26658454, PMID: 16901982, PMID: 15155738). pCDNA3.1 ( + ) Gβ1 was bought at Missouri University of Science and Technology (cdna.org). Plasmids encoding for the following proteins were generously provided as follows: GRK6 and GRK2 by Dr Antonio De Blasi (Istituto Neurologico Mediterraneo Neuromed, Pozzilli, Italy), GRK5 by Dr Robert Lefkowitz (Duke University, Durham, NC). Kir3.2-GFP10 by Dr Terry Hebert (McGill University, Montréal, Canada). Kir3.1 subunit by Dr. Deborah J. Nelson (University of Chicago, Chicago, IL).

### Cell culture and transfection

HEK293 cells were a kind gift of Dr. Laporte, McGill University^57^. They were cultured in 100 mm Petri dishes (Sarstedt, Germany) at 37 °C and 5% CO_2_ in the Dulbecco’s modified Eagle’s medium (DMEM) supplemented with 10% fetal bovine serum, 2 mm l-glutamine and 100 unit mL^−1^ penicillin-streptomycin.

Transient transfections of vectors encoding BRET biosensors in combination with complementary signaling partners were performed in 100 mm Petri dishes (3 × 10^6^ cells) for G protein and Kir3.2 channel activation assays and in 96-wells culture plates coated with polyD-lysine (PerkinElmer, MA, USA) for βarr recruitment assays (32,000 cells/well), using the polyethylenimine transfection reagent (Polysciences, PA, USA) at a PEI/DNA ratio of 3:1^58^. For cAMP production assays, stable cell lines expressing the GFP10-Epac-RlucBRET2-cAMP biosensor^59^ were plated in six-wells plates (Greiner bio-one, Austria) and stably transfected with 1 μg of either MORs or DORs (human or rat) biosensor using PEI. They were selected respectively using hygromycin (100 µg mL^−1^) and puromycin (10 mg mL^−1^).

### BRET assays

Ligand preparation: Agonists were dissolved in DMSO and spotted on 96-well white bottom microplates (Greiner bio-one) using the HP D300 Digital Dispenser (Tecan Life Sciences). DMSO concentration was normalized for each point at 0.334%.

Gαi and Gαo-activation assay: HEK 293 were co-transfected with DOR or MOR (human or rat), either of the BRET biosensors pairs: γ2-GFP10/GαoA-99-RlucII (Ratio Receptor/GFP/RlucII: 1:0.6:0.12), γ2-GFP10/Gαi1–91-RlucII (Ratio Receptor/GFP/RlucII: 1:0.6:0.12), or γ2-GFP10/Gαi2-99-RlucII (Ratio Receptor/GFP/RlucII: 1:0.72:0.12) together with untagged Gβ1(Ratio 1: 0.6)^26^. Forty-eight hours after the transfection, the media was removed and the cells were washed with phosphate-buffered solution (PBS) then re-suspended in PBS + MgCl_2_ (0.429 mM) at a protein concentration ≥0.6 µg µL^−1^. Coelenterazine 400a was added to the cells to a final concentration of 5 µM for 3 min, and 100 µL per well of this mix were subsequently distributed into the 96-well-printed plates. Plates were read 5 min after on the Mithras LB 940 microplate reader (Berthold Technologies, Bad Wildbad, Germany), 3 s per well, with filters set at 400 nm (RlucII) and 515 nm (GFP10); BRET ratios were calculated as GFP10/RlucII emissions. Net BRET values were calculated by subtracting background BRET ratio observed in cells expressing donor G biosensors alone.

Gs activation assay: HEK293 cells stably expressing β2AR were transiently transfected with 200 ng Gαs-67-RlucII, 100 ng Gβ1, and 100 ng GFP10-Gγ1. The day of the experiment, cells were washed with Hank’s balanced salt solution (HBSS) (137 mM NaCl, 5.4 mM KCl, 0.25 mM NaHPO_4_, 0.44 mM KH_2_PO_4_, 1.8 mM CaCl_2_, 0.8 mM MgSO_4_, 4.2 mM NaHCO_3_, pH 7.4) supplemented with 0.1% glucose and 0.1% BSA. Coelenterazine 400a (Coel-400a, Biotium) was added for 5 min to the wells (2.5 μM), then β-adrenergic compounds were added for 4.5 min. BRET was measured and calculated as described above.

Kir 3.2 channel activation assay: HEK 293 were plated onto 100 mm Petri dish and transfected with DOR or MOR (human or rat), the Kir3.2-GFP10/γ2-LucII BRET biosensor pair together with untagged Kir3 channel and G protein subunits^27^ at a ratio of 1:1:0.075:1:0.5, respectively. The BRET assay was performed as described above.

β-arrestin recruitment: HEK 293 cells were co-transfected with sp-FLAG-DOR-GFP10 or sp-FLAG -MOR-GFP10 (human or rat) and βarr1/2-RlucII for β-arrestin1/2 recruitment at a ratio receptor/construct of 1:0.06. Recruitment of βarr2-RlucII was also tested in the presence of, GRK2, GRK5, GRK6 (Ratio receptor/GRK DNA: 1:0.1). Forty-eight hours after transfection, cells were washed with PBS then incubated in Tyrode’s solution (140 mM NaCl, 2.7 mM KCl, 1 mM CaCl_2_, 12 mM NaHCO_3_, 5.6 mM D-glucose, 0.49 mM MgCl_2_, 0.37 mM NaH_2_PO_4_, 25 mM HEPES, pH 7.4) for 30–60 min at 37 °C. Indicated concentrations of agonists, diluted in Tyrode buffer, were added to the wells for 10 min, then cells were incubated for 5 min with Coelenterazine 400a (2.5 µM). BRET2 readings were taken at 37 °C as detailed above. For β2AR β-arrestin2 recruitment, HEK cells were transiently transfected with 50 ng βarr2-RLucII and 300 ng β2AR-GFP10. The day of the experiment, cells were washed with HBSS supplemented with 0.1% glucose and 0.1% BSA. β-adrenergic compounds were added to the wells for 10 min, then coelenterazine 400a was added for 5 min to the wells (2.5 μM). BRET was measured and calculated as described above.

cAMP production assay: Stably-transfected cells expressing the GFP10-Epac-RlucBRET2-cAMP biosensor^59^ and either MORs or DORs were seeded at a density of 30,000 cells/well in a high glucose medium supplemented with 10% newborn calf serum, and grown on 96-well polylysine-coated plates for 48 h. Cells were later transferred to Tyrode buffer and incubated for 15 min at 37 °C. Coelenterazine 400a was then added to a final concentration of 5 µM. Five min later, forskolin (Bioshop, Canada) was introduced (final concentration: 10 µM for rMOR, 15 µM for rDOR, and 25 µM for hMOR and hDOR) followed, 3.5 min later, by increasing concentrations of ligands. BRET2 readings were taken 5 min after ligands were introduced^28^.

### Guinea pig ileum assays

Male Hartley guinea pigs were anesthetized using isoflurane followed by exsanguination. The myenteric plexus of the ileum was dissected according to the method described by Cowie & al.^41^. Briefly, a portion of the ileum was removed (10 cm distal to the cecum) into which a glass rod was inserted. The myenteric plexus was removed from the circular longitudinal muscle via gentle scraping with a moist cotton swab and separated from the muscle using forceps. The resulting myenteric tissue was cut into 2.5 mm strips and placed in oxygenated Krebs buffer (37 °C, gassed with 95% O_2_/5% CO_2_) and tensioned to a baseline tension of 2000 mg. The tissues were washed, equilibrated for 30 min, and subsequently tested for viability with a maximal concentration of Carbachol (300 nM, three times with 10 min of washing, and 10 min of equilibrating in between additions). The final prime was followed by a 20 min wash period followed by a 20 min equilibration period before the start of the experiment. Tissues were continually stimulated with 0.1 Hertz for 1 ms at 20 volts (producing a stimulation equivalent to 80% of the maximal contractile response). Following a 10 min baseline stimulation period, the kappa opioid antagonist nor-binaltorphimine was added (5 nM final) and incubated for 10 min. Finally, cumulative concentration–response curves were generated to each test ligand or vehicle control (DMSO). Isometric tension data (in mg) were collected.

All procedures performed on these animals were in accordance with regulations and established guidelines and were reviewed and approved by Pfizer Institutional Animal Care and Use Committee.

### Curve fitting

Concentration response curves describing ligand responses by different receptors (hMOR, hDOR, rMOR, rDOR, and hb2ADR) were analyzed with Graphpad Prism6, using built-in 3 or 4 parameter logistic equations to obtain independent pEC50 and *E*_max_ values for each receptor–biosensor pair:

1 $$y = a + \left( {b - a} \right)/\left( {1 + 10^{\left( {logEC50 - x} \right) \ast c}} \right)$$

(*y* → measured response; *a* → minimal asymptote, *b* → maximal asymptote; *b − a* → *E*_max_; c → slope).

Concentration response curves were additionally analyzed with the operational model of Black and Leff^32^. As above, curves representing responses elicited by the same receptor at each of the ten different biosensors were fit independently. Fitting was done using Graphpad Prism6 after introducing a set of equations kindly provided by Dr Christopoulos:

*A* = 10$$^x$$

2 $$\begin{array}{l}{\it{operate1}} = \hfill \\ \hfill\left( \left( {1 + A} \right) / \left( { {10^{{\mathrm{Log}}R}} \ast A} \right) \right)^n\hfill \\ \hfill\left( {{\mathrm{used}\, \mathrm{to}\, {\mathrm{fit}}\, {\mathrm{full}}\, {\mathrm{agonists}}}} \right)\end{array}$$

3 $$\begin{array}{l}{\it{operate2}} = \hfill \\ \hfill\left( {\left( {1 + A/{10^{{\mathrm{Log}}KA}}} \right)/\left( {{10^{{\mathrm{Log}}R}}\ast A} \right)} \right)^n\hfill \\ \hfill\left( {{\mathrm{used}}\;{\mathrm{to}}\;{\mathrm{fit}}\;{\mathrm{partial}}\;{\mathrm{agonists}}} \right)\end{array}$$

4 $$\begin{array}{l}{\mathrm{Full}}\;{\mathrm{agonist}} = \hfill \\ \hfill{\mathrm{basal}} + \left( {E_{\mathrm{max}} - {\mathrm{basal}}} \right)/\left( {1 + {\it{operate1}}} \right)\hfill\end{array}$$

5 $$\begin{array}{l}{\mathrm{Partial}}\;{\mathrm{agonist}} = \hfill \\ \hfill{\mathrm{basal}} + \left( {E_{{\mathrm{max}}} - {\mathrm{basal}}} \right)/\left( {1 + {\it{operate2}}} \right)\hfill\end{array}$$

basal → response observed in the absence of agonist; *E*max → maximal response of the system; *n* → slope of the function which links occupancy to response; *KA* → functional affinity (partial agonists); Log(R) → Log(τ/KA).

When using the logistic model, the fits for three and four parameter curves were compared and the best fit taken. If no fitting was possible without constraints, the minimal asymptote was fixed to zero; if this was unsuccessful, the Hill coefficient was additionally fixed to one (i.e.,: only the three parameter fit was considered). If both these constraints proved unsuccessful, and in curves with no inflection point for maximal effect, the highest experimental value was considered *E*_max_. The latter procedure forced the maximal response of very weak partial agonists within the range of experimental data avoiding aberrant predictions due to extrapolation. If no fitting was possible following these constraints, no fitting (NF) status was consigned. If fitting was possible, we made sure that all curves had a Span > 3x SEM, otherwise they were considered as no response (NR).

As used in this study, the operational model does not yield Log(τ) or pKA values for full agonists^25^, which were consigned as not available (NA). In these circumstances, *E*_max_ values were used to differentiate these compounds from partial agonists, and differences among full agonists were established through their consolidated Log(τ/KA) coefficients. It should also be noted that by independently fitting curves for different biosensors, the model does not contemplate interconversion among distinct receptor states stabilized by different effectors.

### Feature reduction, and ligand clustering

Each receptor was represented by a matrix composed of 25 ligands (21 for DORs) × 30 parameters (*E*_max_, Log(τ) and Log(τ/KA) for ten assays). This matrix was created by sampling from the normal distribution around each parameter using the mean and standard deviation thereby incorporating the variance associated with each data point and propagating it through the clustering method. In order to correct for scale differences between parameters, we standardized each column to range between 0 and 1 according to:
  6 $${\mathrm{Standardized}}\;{\mathrm{value}} =\frac{{{X}_{ij}} - {\mathrm{minimum}}_j} {{\mathrm{maximum}}_j - {\mathrm{minimum}}_j}$$
  for every ligand *i* and every parameter *j*.
Process (1) was repeated 1000 times to create 1000 data matrices each independently put through the following procedure (Supplementary Fig. 2).
Nonnegative matrix factorization (NNMF) was used to reduce dimensionality of the data and create the W (ligand * k) and H (k *parameter) basis vectors thereby removing noise and redundancy. We used sparse NNMF to ignore missing data (“NA”, “NF”, and “NR” curves). Difference between the original matrix V and the product of W * H was minimized to less than 1e-7.
K-means clustering was performed on the W basis vector, where the number of clusters equals the number of basis vectors from NNMF (K = k), to assign each compound into a cluster. Note: the phenotypic parameter clusters were obtained using the H vector instead of the W one.
Steps 3 and 4 were repeated 250 times to quantify the fraction of times each compound clustered together resulting in a ligand * ligand frequency matrix ranging from 1 (always clustered together) to 0 (never clustered together). This iterative process quantifies both global and local minima/maxima arising from small variances in clustering resulting from the randomized starting vectors for NNMF and k-means.
(a) The entire process including feature reduction and clustering (3–5) was repeated for different values of k (k = 2 to k = 7), providing a frequency matrix for each k. (b) These six frequency matrices were averaged together to quantify ligand similarity independent of the number of features used as each K may extract unique patterns that may be complementary or orthogonal to results from different K’s.
Steps 3–6 were independently performed on each of the 1000 sampled data matrices providing 1000 composite similarity matrices.
These 1000 matrices were averaged to create a final frequency matrix quantifying how often ligands clustered together and representing total ligand similarity across all concentration response curves.
We visualized the similarity matrix using a dendrogram and a heat map using Orange, created from the distance between each compound in the similarity matrix using a Pearson Correlation.

### Simulation of virtual compounds

We built 16 profiles showing bias and various potencies/efficacies by selecting ranges of KA-τ pairs across six imaginary biosensors. So that our virtual compounds respect these ranges, we invented them by sampling random values of KA and τ within the bounds associated to the imaginary biosensors specified per profile. We used this procedure 20 times for each profile yielding 320 virtual ligands.

As for curve fitting, simulations were conducted under the assumption of independence across biosensors. Using the operational model equation, we generated corresponding concentration response curves (CRC) to which we added 10% noise using the flat distribution. Noisy CRCs were then fitted to both the logistic equation and the operational model equation using the Bayesian inference engine STAN^60^ to yield values of *E*_max_, pEC50, τ, and KA and their associated distributions (from which we computed a standard error of the mean to use in the NNMF pipeline) (values in Supplementary Data 12). The best estimate for τ/KA ratio and its distribution of draws were also computed directly within the fitting process as a transformed parameter.

Selections of subsets of parameter estimations and associated SEM were used in NNMF/k-means clustering. The resulting matrices of frequency of co-occurrences were used to compute distance metrics, hierarchical clustering trees, and tSNE plots whose leaves and data points were colored by profile (Fig. 1, Supplementary Fig. 3, Supplementary Fig. 4).

### Clustering of pharmacological parameters

The 25 (1 per ligand) values for each parameter array (P) were distributed into four smaller arrays corresponding the ligand clusters. We then utilized a two-sample Kolmogorov–Smirnov test to compare each sub-array to the original array (P) to measure if these were randomly sampled from the original array. This provides four *p*-values for each parameter. A significant *p*-value indicates ligands in that cluster are biased toward a specific response for that parameter. This process was repeated for each of the 30 parameters. These *p*-values were then sorted according to (i) the type of parameter considered (e.g., pEC50, *E*_max_, or Log(τ/KA)) or (ii) measurement similarity acquired from the similarity matrix obtained by the NNMF/k-means method detailed above using the H basis vector instead of the W. The procedure is summarized in Supplementary Fig. 7.

### Comparing clusters among complete data sets

To compare clustering similarity between two different data sets we implemented two approaches: (a) Directly comparing the two similarity matrices using pairwise differences and (b) quantifying the overall difference between the two matrices using random simulation to obtain a difference threshold and to establish significance.

Direct comparison: We calculated the difference between every paired value in similarity matrix A and B (representing the similarity between compounds i and j):

7 $${\mathrm{Difference}} = {A_{ij}} - {B_{ij}}$$

The resulting difference matrix is of equal dimensionality to A and B, ranging from 1 (compounds i and j are always clustered together in A but never in B) to −1 (always in B but never in A).

Thresholding and random simulation: We compared the difference in Euclidian distance for every pair of ligands i and j between similarity matrices for data set A and B:

8 $${\mathrm{Difference}} = \left| {\left| {L_{iA} - L_{jA}} \right|} \right| - \left| {\left| {L_{iB} - L_{jB}} \right|} \right|$$

Where L_iA_ and L_jA_ are row vectors representing the similarity of L_i_ and L_j_ to all other ligands in matrix A. We then used random clustering replicates (detailed below) to identify a cutoff value to determine which difference values corresponded to a significant variation between A and B. The final comparison between data set A and B was represented as a proportion:

9 $${\mathrm{Fraction}}\;{\mathrm{change}} = \frac{\# \; significant \;differences}{Total \# \; of\; comparisons}$$

Only ligands tested in both data sets were used (e.g., comparing hMOR to hDOR only used the 21 shared ligands).

Random clustering: For each data set, we created 50 random input data matrices by permuting all mean-standard deviation pairs of data points within the original data matrix. Each random matrix was therefore specific to and equal in size and shape to the original data (e.g., hMOR: (25 * 30); hMOR-βarr: (25 * 15)). We then repeated the entire NNMF/k-means clustering method on each data-shuffled random matrix resulting in 50 random clustering frequency matrices for each data type.

To determine a cutoff value representing significant variation between any two data sets (Difference threshold), we calculated the Euclidian Distance between pairs of compounds in the same cluster and compounds in different clusters for each of the 50 trials. The threshold is the mean value of the overlap between the “same cluster” distribution and the “different cluster” distribution (see Supplementary Fig. 9). Threshold values range between 0.95 and 1.5. Using these thresholds, it was possible to calculate the proportion of significant variation between two matrices. To quantify if this change was significant, we calculated the fraction of significant changes (using thresholds) between the clustering from the 50 randomized data sets (e.g., 50 random hDOR) compared with reference cluster (e.g., hMOR). The resulting distribution of 50 values represented the proportion of random changes from the reference. This distribution was used to calculate a *z*-score for the difference value of the actual data (hMOR vs hDOR):

10 $${\mathrm{z}} {{-}} {\mathrm{score}} = \frac{{( {{\mathrm{fraction}}\;{\mathrm{change}}\;{\mathrm{in}}\;{\mathrm{actual}}\;{\mathrm{data}}} ) - ( {{\mathrm{mean}}\;{\mathrm{random}}\;{\mathrm{change}}} )}} {( {{\mathrm{STD}}\;{\mathrm{random}}\;{\mathrm{changes}}} )}$$

### Comparing clusters generated with complete data and subsets

In order to calculate whether clustering from data subset I (e.g., hMOR-Barr) changed more than data subset J (e.g., hMOR-G protein) compared with the complete data set clusters (e.g., hMOR), we compared the 50 random similarity matrices to the reference (e.g., hMOR) and calculated the fraction of significant changes using the method detailed above. As a result, we obtained an array of 50 values representing the random change from reference. This array was created for both subsets (e.g., hMOR-βarr and hMOR-G protein). We then iteratively, with replacement, randomly sampled 1 value from each of these arrays and calculated the difference to create a distribution of 1000 values indicating the random expected difference between these two subsets of data. We then calculated a *z*-score using the mean and standard deviation of this distribution and the actual observed difference.

### Clustering ligands according to structural similarities

Each ligand was represented using three standard fingerprint representations: (ECFP-6) Extended-Connectivity Fingerprints (ECFPs) (http://accelrys.com/products/collaborative-science/biovia-pipeline-pilot/), Functional-Class Fingerprints (FCFPs) and MDL MACCS keys. A similarity matrices for each different fingerprint was generated for the 25 ligands in the data set, where each value in the matrix (S_i,j_) corresponds to the Tanimoto similarity value between compound *i* and compound *j* and ranges from a value 0 to 1 (1 being most similar). We combined these three matrices into a single matrix of dimensions (25 compounds × 75 comparisons), and repeated the NNMF/k-means clustering algorithm on the data to yield a structural similarity matrix.

### Correlating signaling data to side effect report frequency

A list of all MOR-active compounds was created by searching DrugBank for all approved drugs which activate MOR. The resulting list was intersected with the list of drugs in the FDA’s Adverse Event Reporting System data for which a standardized gamma (SD gamma) score could be generated at the preferred term (PT) level according to the method of Johnson et al.^33^. Briefly, SD gamma scoring is a statistical approach to identify disproportionately high, or low, numbers of drug-event occurrences by normalizing for number of drugs and number of event reports. SD gamma scores for each event were averaged for all resulting MOR compounds, and PT events were sorted by average score to produce a listing of high-scoring events most clinically relevant to opioid therapy (80 highest scores were considered). A similar procedure was completed to find the 80 side effects associated with β2ADR-active compounds.

Individual drug SD gamma scores for frequently reported events were then correlated to Euclidian distances separating prescription opioids (tramadol, buprenorphine, oxycodone, morphine, fentanyl, and loperamide) in hMOR and structural similarity matrices. SD gamma scores were additionally correlated to transduction coefficients for BRET or guinea pig contractility responses respectively normalized to Met-ENK (∆Log(τ/KA)_MET_) or loperamide (∆Log(τ/KA)_LOP_). Note that LOP and Met-ENK are balanced ligands that co-cluster in every data set such that differences due to normalization are simply scalar. Individual drug SD gamma scores clinically prescribed β2ADR ligands used in the study (isoproterenol, norepinephrine, salbutamol, salmeterol, pindolol, carvedilol, and metoprolol) were similarly correlated to Euclidian distances separating these ligands in the β2ADR similarity matrix or to transduction coefficients for BRET responses where isoproterenol was the standard.

### Statistical analysis

Correlation analysis: GraphPad Prism6 was used to evaluate correlation between drug distance in cluster and the frequency of reports of adverse events.

All statistical comparisons were two-sided except when contrasting partial and whole similarity matrices.

### Reporting summary

Further information on research design is available in the Nature Research Reporting Summary linked to this article.

## Supplementary information

Supplementary Information

Description of Additional Supplementary Files

Supplementary Data 1

Supplementary Data 2

Supplementary Data 3

Supplementary Data 4

Supplementary Data 5

Supplementary Data 6

Supplementary Data 7

Supplementary Data 8

Supplementary Data 9

Supplementary Data 10

Supplementary Data 11

Supplementary Data 12

Reporting Summary

## Data Availability

All data generated or analyzed in this study are included in the article and supplementary materials or provided as source data files.

## Source data

Source Data

## Code availability

All clustering and cluster comparisons were conducted using Python 2.7.6. Complete source code is available for download at http://github.com/JonathanGallion/Benredjem-Gallion.
