# Author Correction: Exploring use of unsupervised clustering to associate signaling profiles of GPCR ligands to clinical response

**DOI:** 10.1038/s41467-020-15945-y

**Published:** 2020-04-16

**Authors:** Besma Benredjem, Jonathan Gallion, Dennis Pelletier, Paul Dallaire, Johanie Charbonneau, Darren Cawkill, Karim Nagi, Mark Gosink, Viktoryia Lukasheva, Stephen Jenkinson, Yong Ren, Christopher Somps, Brigitte Murat, Emma Van Der Westhuizen, Christian Le Gouill, Olivier Lichtarge, Anne Schmidt, Michel Bouvier, Graciela Pineyro

**Affiliations:** 1 Department of Pharmacology and Physiology, Faculty of Medicine, Université de Montréal, Montréal, QC H3T 1J4 Canada; 2 CHU Sainte-Justine Research Center, Montréal, QC H3T 1C5 Canada; 3 Baylor College of Medicine, Houston, TX 77030 USA; 4 Pfizer Inc, Groton, CT 06340 USA; 5 College of Medicine, Member of QU Health, Qatar University, Doha, Qatar; 6 Institute for Research in Immunology and Cancer, Department of Biochemistry and Molecular Medicine, Université de Montréal, Montréal, QC H3T 1J4 Canada; 7 Present Address: Apollo Therapeutics LLP, Stevenage Bioscience Catalyst, Gunnels Wood Road, Stevenage, SG1 2FX UK; 8 Present Address: Pfizer Inc, La Jolla, CA 92121 USA; 9 Present Address: Decibel Therapeutics, 1325 Boylston Street, Boston, MA 02215 USA; 10 Present Address: Monash Institute of Pharmaceutical Sciences, Parkville, VIC 3052 Australia

**Keywords:** Computational biology and bioinformatics, Drug screening, Pharmacology

Correction to: *Nature Communications* 10.1038/s41467-019-11875-6, published online 9 September 2019.

The original version of this Article omitted the following from the Acknowledgements:

This study was supported by the Quebec Consortium on Adverse Effects of Pain Medications, an initiative funded by the Quebec Pain Research Network (QPRN) of the Fonds de recherche du Québec Santé.

This has now been corrected in both the PDF and HTML versions of the Article.

