# Supplementary material for: Exploring use of unsupervised clustering to associate signaling profiles of GPCR ligands to clinical response
Source: Nat Commun. 2019 Sep 9;10:4075. doi: 10.1038/s41467-019-11875-6 (PMC6733853; doi:10.1038/s41467-019-11875-6)
Supplement: Supplementary file 1 — Supplementary Information [file 41467_2019_11875_MOESM1_ESM.pdf]

“EXPLORING USE OF UNSUPERVISED CLUSTERING TO ASSOCIATE SIGNALING PROFILES OF GPCR LIGANDS TO CLINICAL RESPONSE”

Benredjem et al.

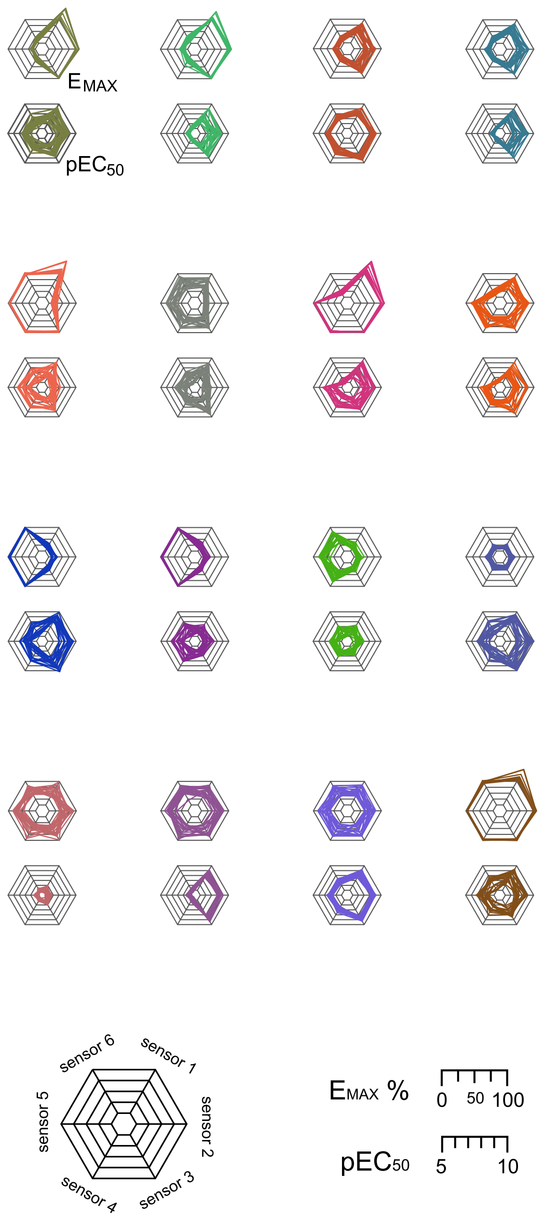

E_{MAX}
pEC_{50}
sensor 6
sensor 1
sensor 5
sensor 2
sensor 4
sensor 3
E_{MAX} %
0
50
100
pEC_{50}
5
10

Supplementary Figure 1. Profiles of virtual compounds classed by Emax average distance. Each profile is shown as a pair of stacked radar plots giving Emax (top) and pEC50 (bottom) for 6 readouts (sensors 1 to 6). Emax and pEC50 radar plot overlays of the 20 compounds in each profile (see materials and methods) arranged in a 4 x 4 array and loosely grouped as per inter-profile average Euclidian distance of Emax (normalized sensor-wise).

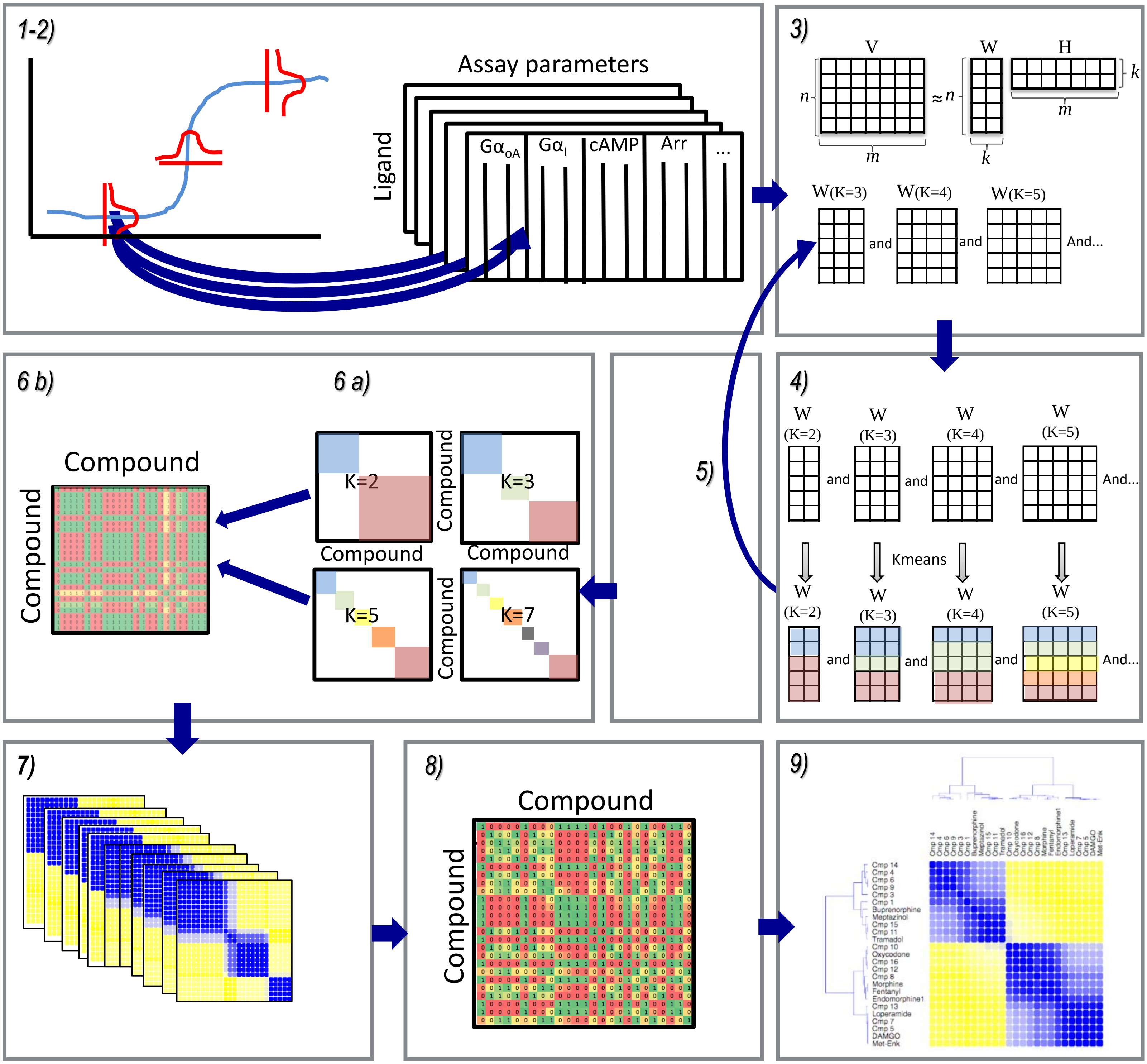

1-2)
Assay parameters
Ligand
Gα$_{oA}$
Gα$_{i}$
cAMP
Arr
...
3)
V
W
H
n
≈ n
m
k
k
m
W(K=3)
W(K=4)
W(K=5)
and
and
And...
4)
W (K=2)
W (K=3)
W (K=4)
W (K=5)
and
and
and
And...
Kmeans
W (K=2)
W (K=3)
W (K=4)
W (K=5)
and
and
and
And...
5)
6 b)
6 a)
Compound
Compound
K=2
K=3
K=5
K=7
Compound
Compound
Compound
Compound
7)
8)
Compound
Compound
9)

Supplementary Figure 2. Stepwise method used to cluster hMOR ligands according to pharmacodynamic parameters. 1-2) To incorporate the variance associated with the value of each parameter (pLog(τ), Emax, Log(τ/KA) describing the mean concentration response curve for each ligand at distinct biosensors we iteratively sampled from the normal distribution centered at the parameter's mean and dispersed according to its standard deviation, to produce 1000 sampled data matrices. This procedure thereby propagates the variation associated with each mean parameter value. ***3)*** NNMF is independently performed on each sampled matrix to reduce the dimensionality of the data prior to clustering, yielding W (ligand) and H (parameter) basis vectors (*upper panel*); NNMF is repeated for (k=2 to k=7) (*lower panel*). ***4)*** Each individual basis vector (K=2-7) is then clustered by K-means. ***5)*** The NNMF and K-means process is repeated 250 times for each value of K and ***6)*** *a)* For each K (2-7), a ligand similarity matrix is derived, indicating co-clustering frequency of ligands i and j in the 250 repeats; *b)* Frequency matrices k=2 through k=7 are averaged into a 'composite similarity matrix' such that each of the 1000 original data samples generates a matrix combining information obtained through 2-7 dimensions, and therefore independent of k. ***7)*** The 'composite similarity matrix' produced from each of the 1000 samples are averaged to create ***8)*** a final ligand similarity matrix quantifying clustering frequency over all 1000 iterations ***9)*** which is then represented as a heat map and a dendrogram.

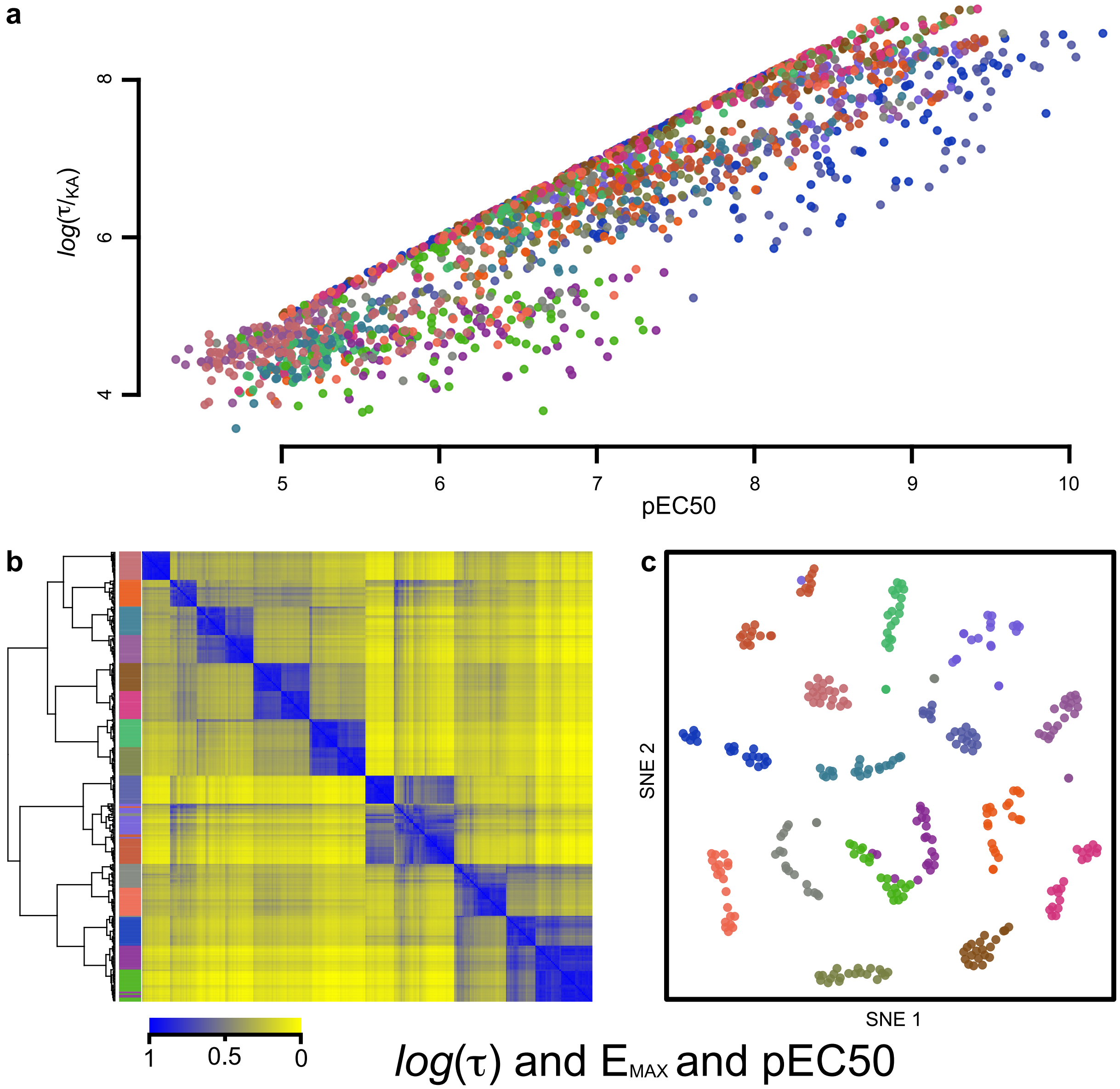

log(τ) and E_{MAX} and pEC50

Supplementary Figure 3. Log(τ/KA) and pEC50 values are correlated but not congruent. Correlation between Log(τ/KA) and pEC50 values generated for the 320 virtual compounds across the 6 readouts (r^2=0.84) **(a).** Indicated parameters were subject to NNMF followed by k-means clustering to produce similarity matrices represented as a heat map/dendrogram pair **(b)** and a t-SNE plot (both computed as described in Fig. 1 **(c)**. Ligands were colour-coded according to their profile.

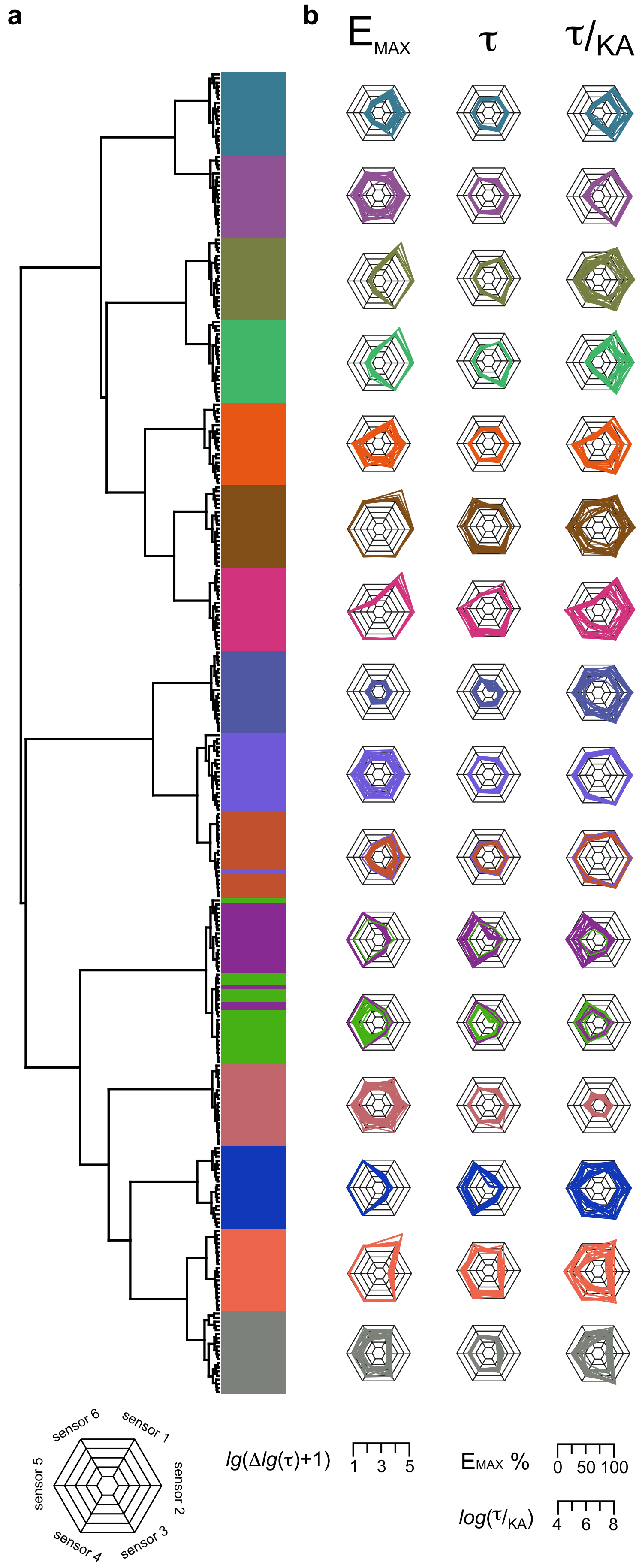
a
b
EMAX
τ
τ/KA
sensor 1
sensor 2
sensor 3
sensor 4
sensor 5
sensor 6
lg(Δlg(τ)+1)
1 3 5
EMAX %
0 50 100
log(τ/KA)
4 6 8

Supplementary Figure 4. Graphic representation of parameters defining virtual compounds clustered according to similarities in Log(τ), Emax and Log(τ/KA). Hierarchical clustering tree (function hclust in R using the ward.D2 metric) with leaf coloured by profile shows the separation of virtual ligands from different profiles (materials and methods). Cuts in the tree essentially coincide with the profiles **(a)**. Radar graphs were computed for each cluster and each parameter considered in the NNMF/k-means clustering showing that group assigenment sometimes depends on subtle variations in any of the selected parameters. Radar plot axes are chosen as to best represent the variation in parameter values. Emax has linear axes. We capture the large dynamic range in τ while still emphasising variation at the pivot value of 1.0, as $\log_2$ fold changes relative to the minimum observed value. On this scale, τ values of 0.1, 1 and 10 are about 2.1, 2.9 and 3.4 respectively (the change between successive grid levels in τ value is $2^{2^i} / 2^{2^{(i-1)}}$, or: 2x, 4x, 16x, 256x, 65536x. These are the multipliers of their previous grid level's value; ie: grid level 3 represents a τ value that is 16 times as large as that represented at grid level 2. τ/KA values are expressed as $\log_{10}$ **(b)**.

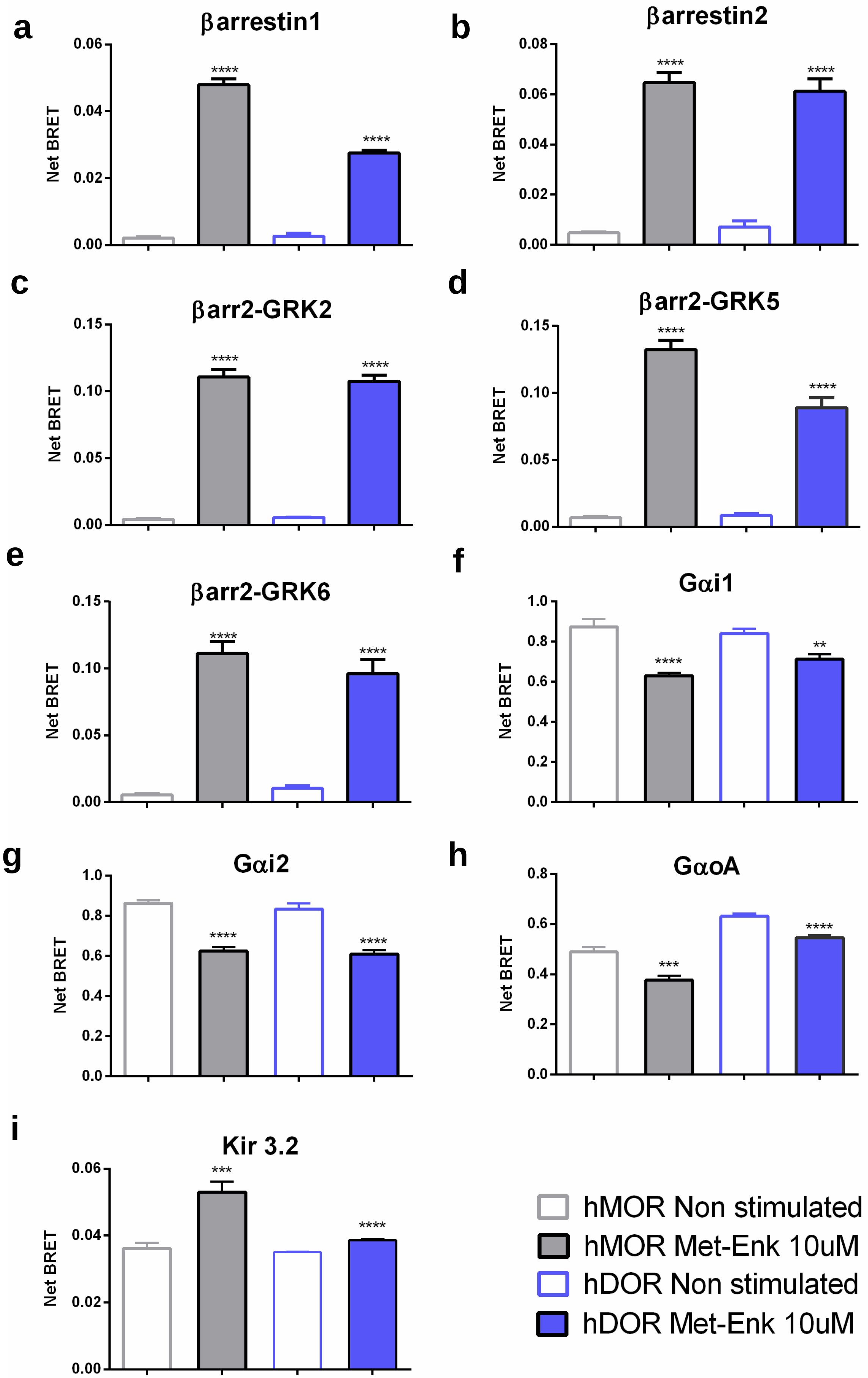

a
βarrestin1
b
βarrestin2
c
βarr2-GRK2
d
βarr2-GRK5
e
βarr2-GRK6
f
Gαi1
g
Gαi2
h
GαoA
i
Kir 3.2
Net BRET
hMOR Non stimulated
hMOR Met-Enk 10uM
hDOR Non stimulated
hDOR Met-Enk 10uM

Supplementary Figure 5. net BRET values of hMOR and hDOR responses obtained with different biosensors. Net BRET values obtained in single point BRET assays monitoring hMOR (green) and hDOR (blue) responses across indicated bi-molecular biosensors are represented as bar graphs for: **(a)** βarr1 recruitment, **(b)** βarr2 recruitment, **(c)** βarr2 recruitment in presence of GRK2, **(d)** βarr2 recruitment in presence of GRK5, **(e)** βarr2 recruitment in presence of GRK6, **(f)** Gαi1 activation, **(g)** Gαi2 activation, **(h)** GαoA activation and **(i)** Kir3.1/3.2 activation. Each response was tested in the presence (10μM; *filled bar*) or absence (*empty* bar) of Met-Enkephalin (Met-ENK) and corresponds to mean ± SEM of 9-12 independent experiments. Results were analyzed using non-paired t-test. ** $p \leq 0.01$; *** $p \leq 0.001$; **** $p \leq 0.0001$.

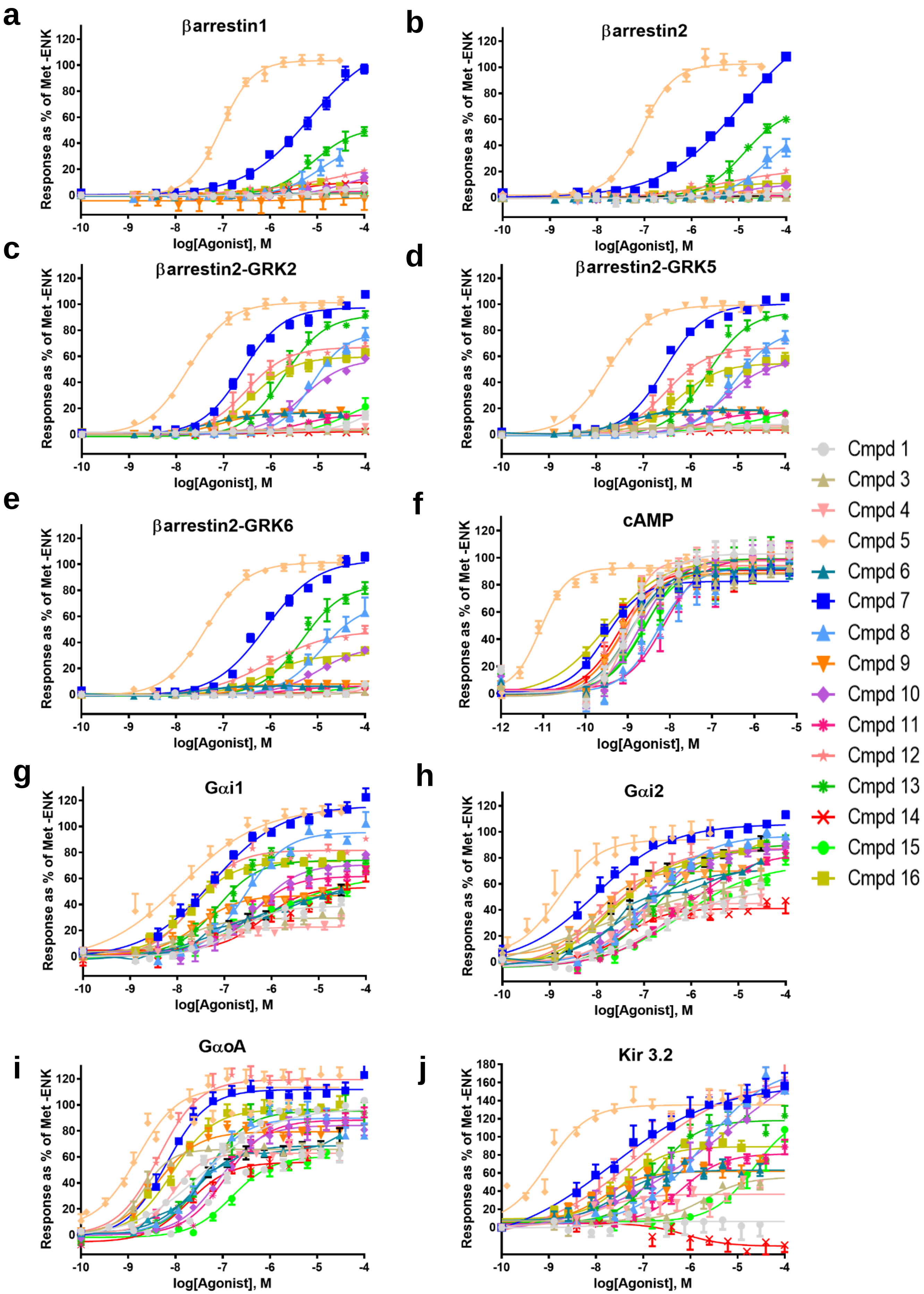
a
βarrestin1
b
βarrestin2
c
βarrestin2-GRK2
d
βarrestin2-GRK5
e
βarrestin2-GRK6
f
cAMP
g
Gαi1
h
Gαi2
i
GαoA
j
Kir 3.2
Response as % of Met -ENK
log[Agonist], M
Cmpd 1
Cmpd 3
Cmpd 4
Cmpd 5
Cmpd 6
Cmpd 7
Cmpd 8
Cmpd 9
Cmpd 10
Cmpd 11
Cmpd 12
Cmpd 13
Cmpd 14
Cmpd 15
Cmpd 16

Supplementary Figure 6. βarr recruitment and G protein responses by novel opioid ligands were monitored using BRET-based biosensors. Novel hMOR ligands were identified in a screening campaign carried out at Pfizer Inc. to identify MOR agonists with poor or no βarr recruitment. A sample of those identified were tested using BRET-based biosensors to monitor response for : **(a)** βarr1 recruitment, **(b)** βarr2 recruitment, **(c)** βarr2 recruitment in presence of GRK2, **(d)** βarr2 recruitment in presence of GRK5, **(e)** βarr2 recruitment in presence of GRK6, **(f)** cAMP, **(g)** Gαi1 activation, **(h)** Gαi2 activation**, (i)** GαoA activation and **(j)** Kir3.1/3.2 activation. Results correspond to mean ± SEM of 3-12 independent experiments. Curves were normalized to the maximal effect produced by Met-ENK, which was tested in all experimental runs (n = 16-29). Curves were fit with operational model and logistic equations (curves shown were fit with the logistic model; parameters from both fits provided in Supplementary Data 1).

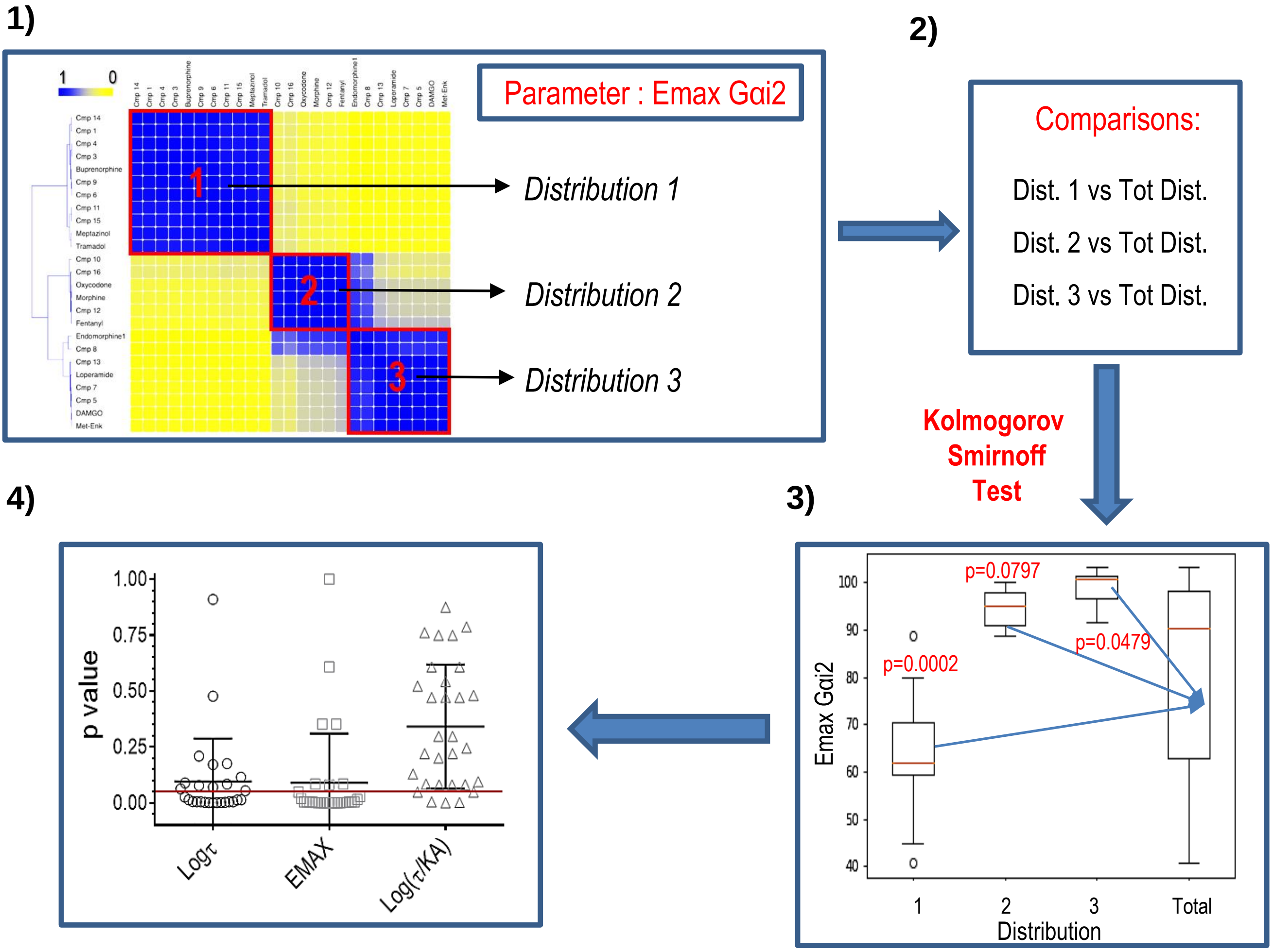
1)
Parameter : Emax Gαi2
1
0
Cmp 14
Cmp 1
Cmp 4
Cmp 3
Buprenorphine
Cmp 9
Cmp 6
Cmp 11
Cmp 15
Meptazinol
Tramadol
Cmp 10
Cmp 16
Oxycodone
Morphine
Cmp 12
Fentanyl
Endomorphine1
Cmp 8
Cmp 13
Loperamide
Cmp 7
Cmp 5
DAMGO
Met-Enk
1
2
3
Distribution 1
Distribution 2
Distribution 3
2)
Comparisons:
Dist. 1 vs Tot Dist.
Dist. 2 vs Tot Dist.
Dist. 3 vs Tot Dist.
Kolmogorov Smirnoff Test
3)
Emax Gαi2
100
90
80
70
60
50
40
p=0.0002
p=0.0797
p=0.0479
1
2
3
Total
Distribution
4)
p value
1.00
0.75
0.50
0.25
0.00
Logτ
EMAX
Log(τ/KA)

Supplementary Figure 7. Stepwise method used to determine parameter contribution to ligand segregation into functional clusters. 1) We measured how well each of the 30 parameters considered individually differentiated the three ligand clusters. This was calculated by identifying which of the parameters (e.g. Gαi2 Emax) showed statistical difference across the compound clusters. Practically, we grouped the Gαi2 Emax values for compounds in each cluster (1, 2, and 3) and **2)** measured if they represented biased selections from the total distribution (all values for 25 ligands combined) using a Kolmogorov Smirnoff. ***3)*** Comparisons for each parameter provides a group of 3 p values. ***4)*** The procedure is repeated for each of the 30 parameters to generate corresponding p values that are then plotted according to parameter type or according to similarity in relative magnitudes (shown in figure 3c-d).

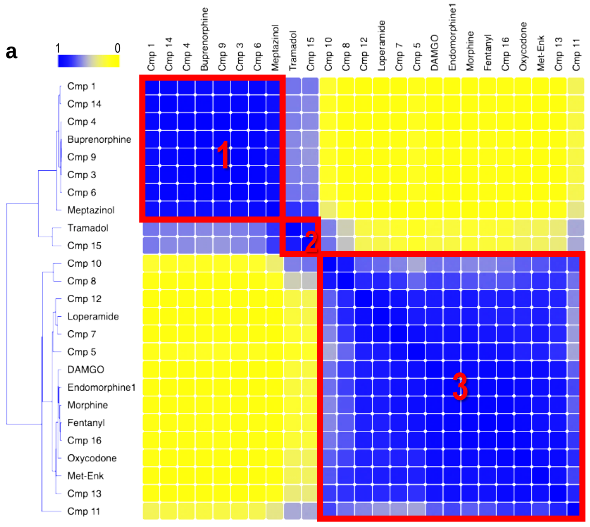

G protein hMOR

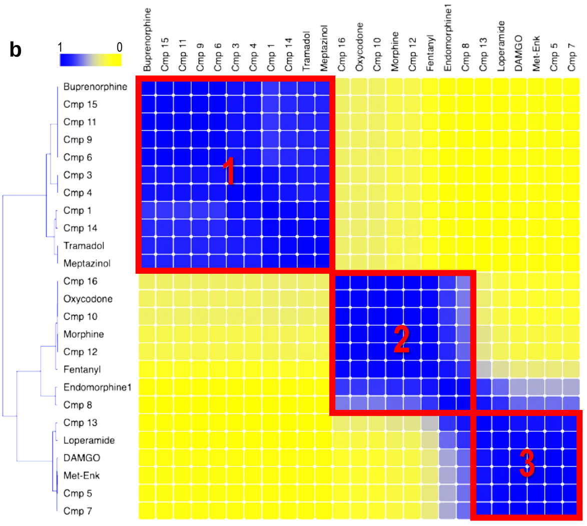

βarrestin hMOR

Supplementary Figure 8. Heat maps and cluster assignements obtained using partial hMOR datasets for βarr or G protein parameters. Shown are heat maps and clusters generated by NNMF and k-means analysis of partial datasets for all G protein mediated-responses ***(a)*** and all βarr recruitment readouts monitored by BRET ***(b)***. Blue (1) and yellow (0) respectively indicate consistent assignement of pairs of ligands to the same or different clusters.

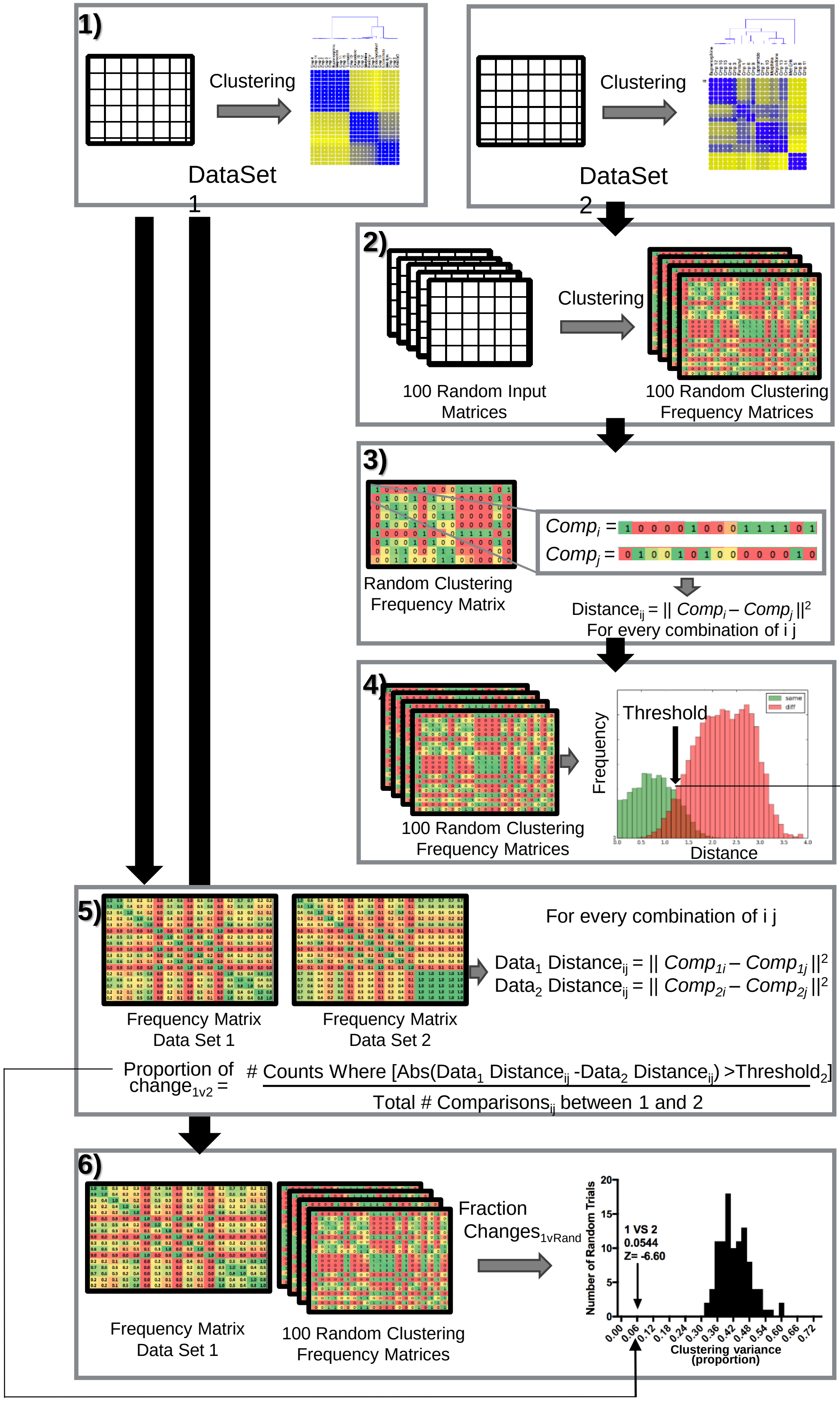

1)
Clustering
DataSet 1
Clustering
DataSet 2
2)
Clustering
100 Random Input Matrices
100 Random Clustering Frequency Matrices
3)
Random Clustering Frequency Matrix
Comp_i =
Comp_j =
Distance_ij = || Comp_i – Comp_j ||^2
For every combination of i j
4)
Threshold
Frequency
Distance
same
diff
100 Random Clustering Frequency Matrices
5)
Frequency Matrix Data Set 1
Frequency Matrix Data Set 2
For every combination of i j
Data_1 Distance_ij = || Comp_1i – Comp_1j ||^2
Data_2 Distance_ij = || Comp_2i – Comp_2j ||^2
Proportion of change_1v2 =
Counts Where [Abs(Data_1 Distance_ij -Data_2 Distance_ij) >Threshold_2]
Total # Comparisons_ij between 1 and 2
6)
Frequency Matrix Data Set 1
100 Random Clustering Frequency Matrices
Fraction Changes_1vRand
Number of Random Trials
1 VS 2
0.0544
Z= -6.60
Clustering variance (proportion)

Supplementary Figure 9*. *Stepwise comparison of clusters produced with different datasets. 1) Use clustering methodology to obtain frequency matrix and cluster assignements for both data sets of interest; e.g. set of parameters for all responses tested for hMOR (dataset 1) vs partial dataset for parameters describing βarr responses at the receptor (dataset 2). ***2)*** Create 100 randomized matrices of dataset 2 by permuting the input values to create a matrix of equal size but shuffled values. Then perform NNMF/k-means analysis on each shuffled matrix resulting in 100 random frequency matrices. ***3)*** For each of the 100 frequency matrices of randomized data, calculate the Euclidian distance between $Comp_i$ and $Comp_j$. Sort distance values based on whether the compounds (i and j) are in the same cluster or different clusters to produce a distribution of distances as shown in next step. ***4)*** Determine a threshold value separating the distribution of distances between compounds in the same cluster and the distances between compounds in different clusters***. 5)*** Calculate and compare the $distance_{ij}$ for dataset 1 vs the $distance_{ij}$ for dataset 2. The proportion of differences between the two matrices greater than the threshold value represents variance in clustering. ***6)*** Finally, measure the clustering similarity of data set 1 vs the 100 random matrices of dataset 2 to obtain a distribution of proportions greater than the threshold distance; this proportion describes random variance. Use z-score to quantify if the proportion of differences describing variance between dataset 1 and the actual dataset 2 is statistically different from the proportion describing random variance.

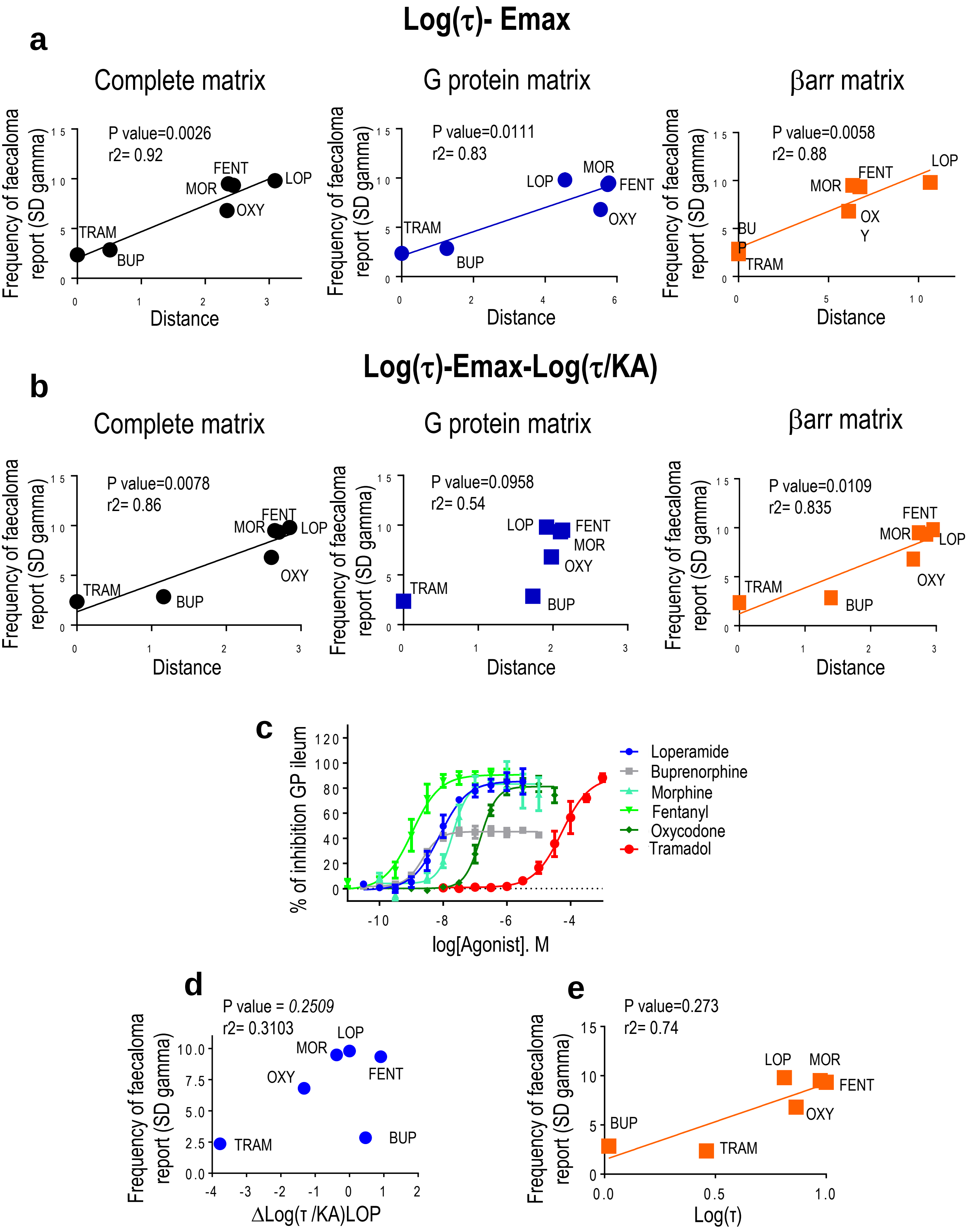
a
Log(τ)- Emax
Complete matrix
G protein matrix
βarr matrix
P value=0.0026
r2= 0.92
P value=0.0111
r2= 0.83
P value=0.0058
r2= 0.88
Frequency of faecaloma report (SD gamma)
Distance
TRAM
BUP
MOR
FENT
LOP
OXY
b
Log(τ)-Emax-Log(τ/KA)
Complete matrix
G protein matrix
βarr matrix
P value=0.0078
r2= 0.86
P value=0.0958
r2= 0.54
P value=0.0109
r2= 0.835
c
% of inhibition GP ileum
log[Agonist]. M
Loperamide
Buprenorphine
Morphine
Fentanyl
Oxycodone
Tramadol
d
P value = 0.2509
r2= 0.3103
ΔLog(τ /KA)LOP
e
P value=0.273
r2= 0.74
Log(τ)

Supplementary Figure 10. Correlating specific signals to reported opioid side effects. Partial matrices in which drugs were classified according to G protein or βarr responses were generated using either Log(τ)-Emax ***(a)*** or Log(τ)-Emax-Log(τ/KA) ***(b)*** as classification criteria. Distances separating ligands in partial and complete matrices were consigned, and correlated to frequency of faecaloma report as indicated in the figure. Inhibitory effect of standard opioid ligands on contractility of guinea pig ileum were assessed ***(c)*** Normalized transduction coefficients ($\Delta Log(\tau/KA)_{LOP}$) ***(d)*** and operational efficacy (Log(τ)) values ***(e)*** were correlated to corresponding SD gamma scores for faecaloma report.

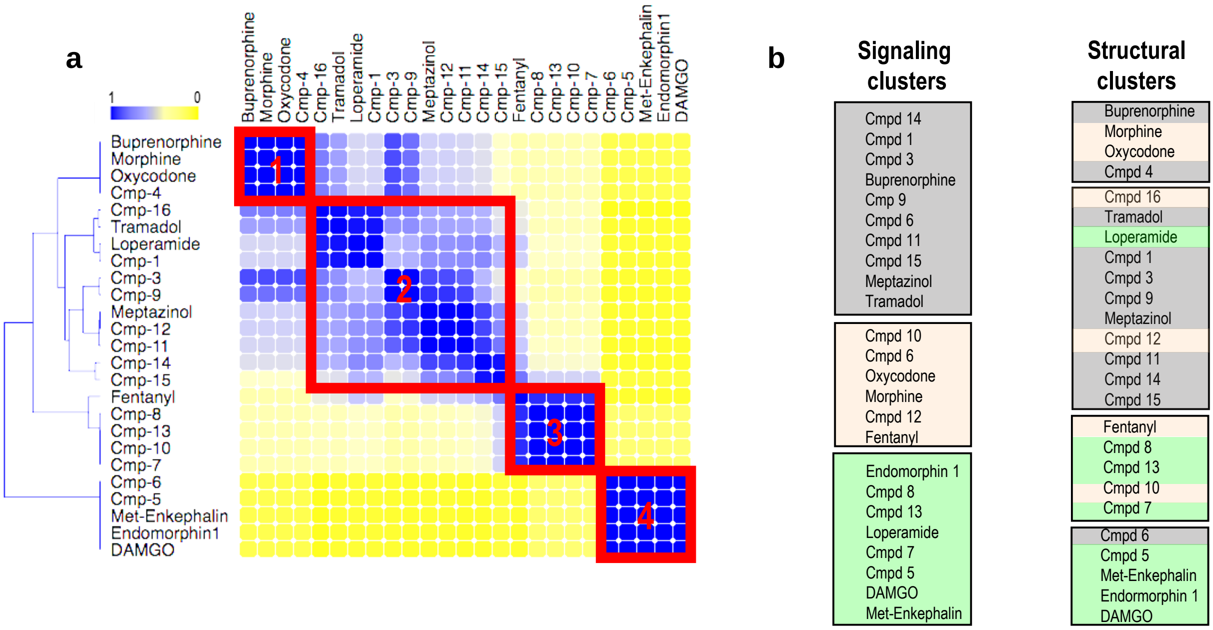
a
1
0
Buprenorphine
Morphine
Oxycodone
Cmp-4
Cmp-16
Tramadol
Loperamide
Cmp-1
Cmp-3
Cmp-9
Meptazinol
Cmp-12
Cmp-11
Cmp-14
Cmp-15
Fentanyl
Cmp-8
Cmp-13
Cmp-10
Cmp-7
Cmp-6
Cmp-5
Met-Enkephalin
Endomorphin1
DAMGO
1
2
3
4
b
Signaling clusters
Cmpd 14
Cmpd 1
Cmpd 3
Buprenorphine
Cmp 9
Cmpd 6
Cmpd 11
Cmpd 15
Meptazinol
Tramadol
Cmpd 10
Cmpd 6
Oxycodone
Morphine
Cmpd 12
Fentanyl
Endomorphin 1
Cmpd 8
Cmpd 13
Loperamide
Cmpd 7
Cmpd 5
DAMGO
Met-Enkephalin
Structural clusters
Buprenorphine
Morphine
Oxycodone
Cmpd 4
Cmpd 16
Tramadol
Loperamide
Cmpd 1
Cmpd 3
Cmpd 9
Meptazinol
Cmpd 12
Cmpd 11
Cmpd 14
Cmpd 15
Fentanyl
Cmpd 8
Cmpd 13
Cmpd 10
Cmpd 7
Cmpd 6
Cmpd 5
Met-Enkephalin
Endormorphin 1
DAMGO

Supplementary Figure 11. Structural and signaling clusters display non-random similarity. Shown is the similarity heat map and clusters generated by NNMF and k-means analysis of 75 Tanimoto similarity values describing opioid ligand resemblance according to three different structural footprints (ECFP-6; FCFP-6; MDL MACCS). Yellow and blue respectively indicate ligands that never or always cluster together ***(a)***. Graphical comparison of cluster composition in functional and structural matrices. Colours show how ligands in the three signaling clusters redistribute when classified using structural criteria ***(b)***.

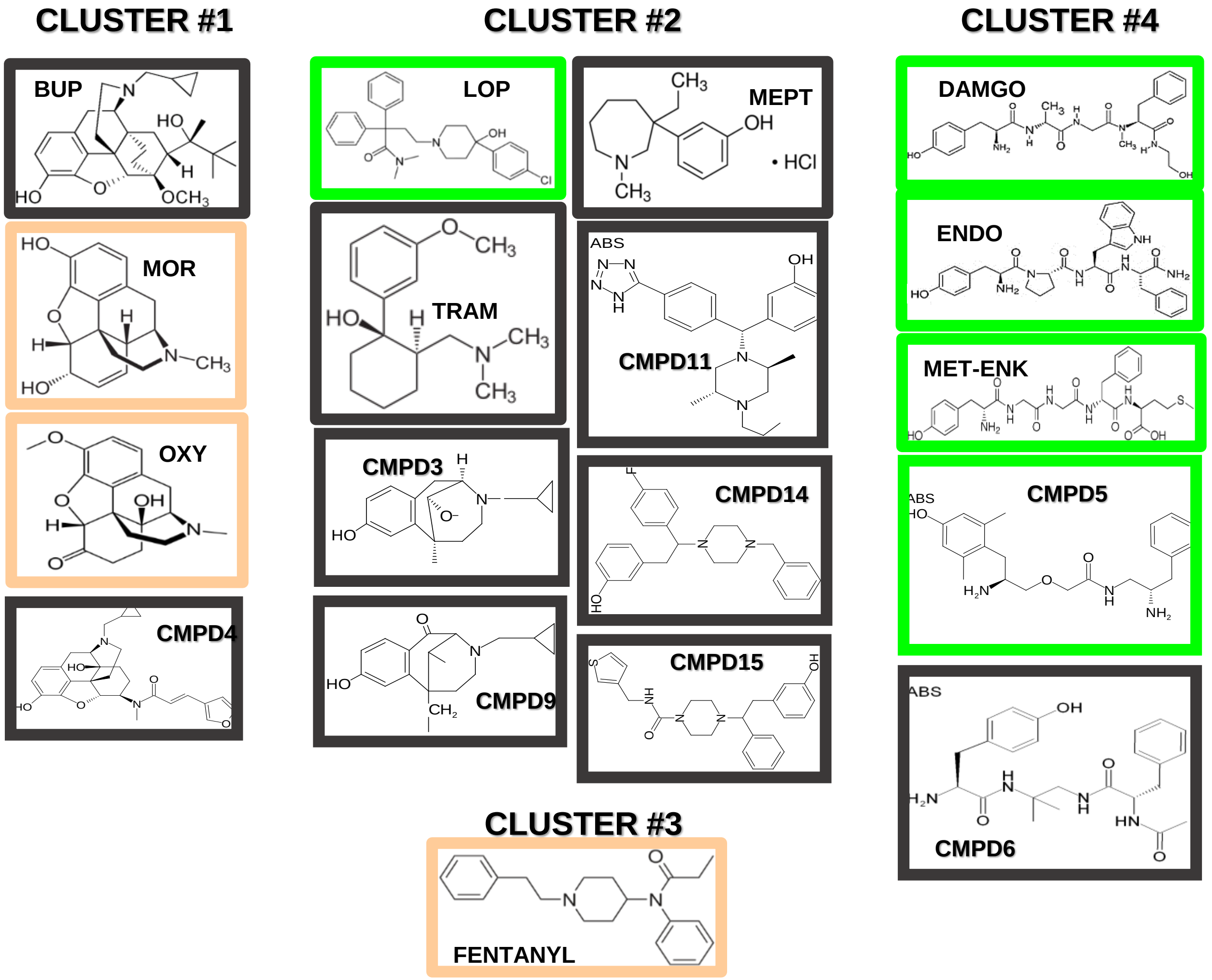
CLUSTER #1
BUP
MOR
OXY
CMPD4
CLUSTER #2
LOP
MEPT
TRAM
CMPD11
CMPD3
CMPD14
CMPD9
CMPD15
CLUSTER #3
FENTANYL
CLUSTER #4
DAMGO
ENDO
MET-ENK
CMPD5
CMPD6

Supplementary Figure 12. Chemical structures for standard and novel opioid ligands. Ligand structures are ordered according to the categories established by clustering Tanimoto similarity values. Either novel compounds, standard ligands or both are provided as representatives of each category. Frames for the different structures are color coded to represent their standing in functional clusters as follows: cluster #1 gray; cluster #2 peach; cluster #3 light green.

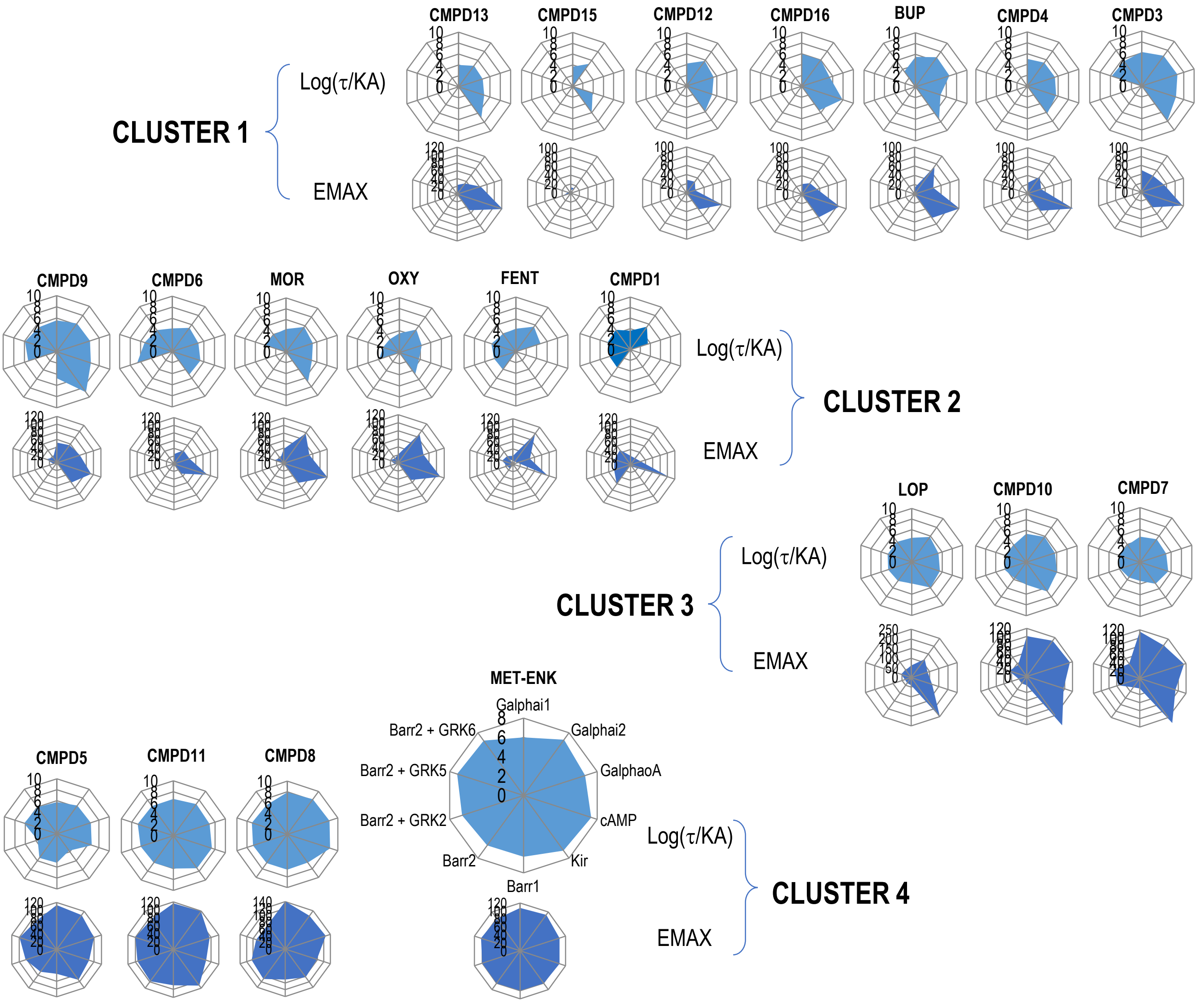
CLUSTER 1
Log(τ/KA)
EMAX
CMPD13
CMPD15
CMPD12
CMPD16
BUP
CMPD4
CMPD3
CLUSTER 2
CMPD9
CMPD6
MOR
OXY
FENT
CMPD1
Log(τ/KA)
EMAX
CLUSTER 3
Log(τ/KA)
EMAX
LOP
CMPD10
CMPD7
CLUSTER 4
CMPD5
CMPD11
CMPD8
MET-ENK
Galphai1
Galphai2
GalphaoA
cAMP
Kir
Barr1
Barr2
Barr2 + GRK2
Barr2 + GRK5
Barr2 + GRK6
Log(τ/KA)
EMAX

Supplementary Figure 13. Graphic representation of pharmacodynamic parameters of hDOR ligands in different clusters. Radial graphs were used to represent operational transduction coefficients (Log(τ/KA)) and logistic Emax values. Each radius corresponds to the magnitude of Log(τ/KA) or Emax values. Transduction coefficients are in logarithmic scale, Emax values were normalized to maximal Met-ENK response, and are presented on linear scale. The key specified only for Met-ENK applies to all radial graphs and shows the order in which information for each biosensor is provided.

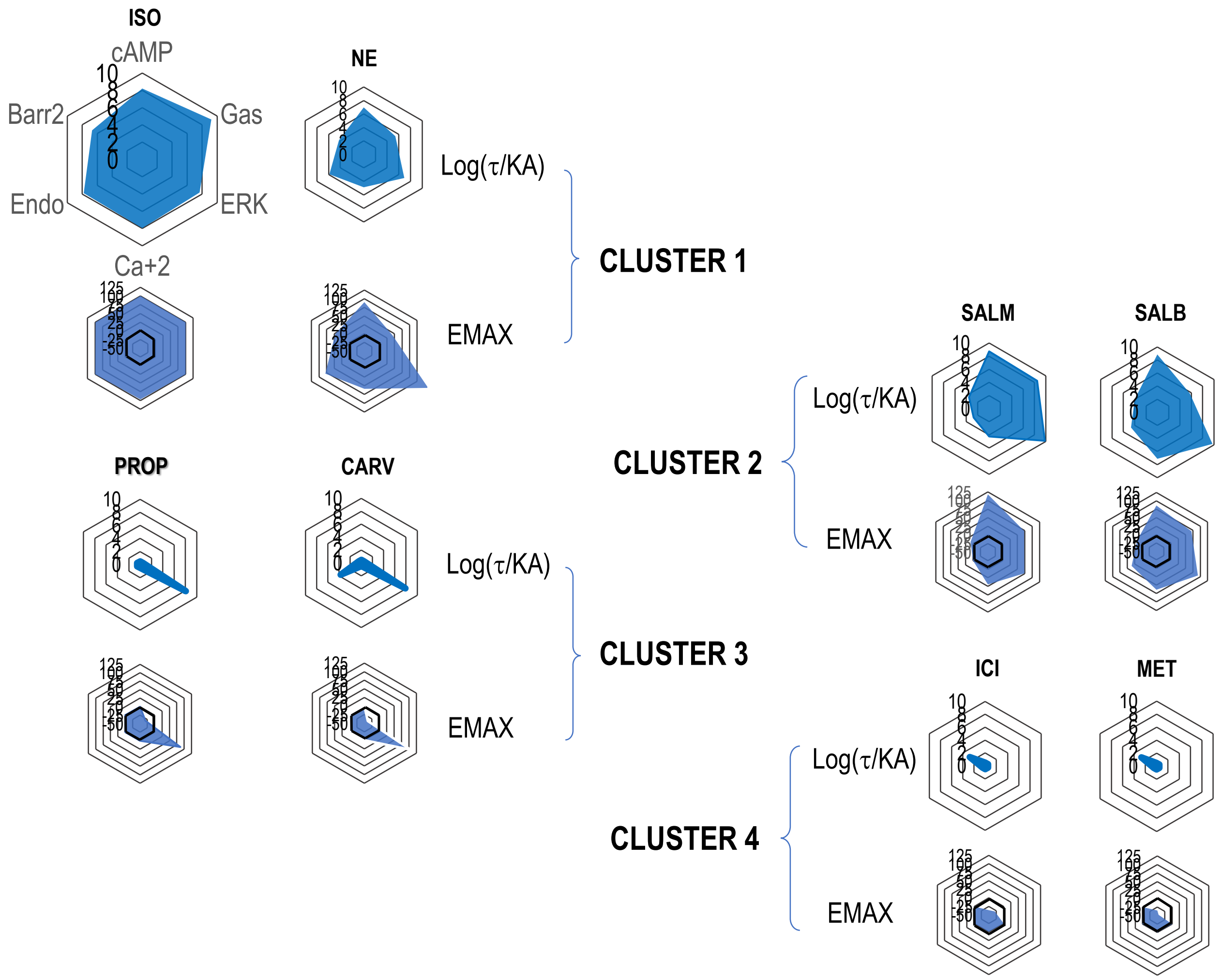
ISO
cAMP
Gas
ERK
Ca+2
Endo
Barr2
NE
Log(τ/KA)
EMAX
CLUSTER 1
PROP
CARV
Log(τ/KA)
EMAX
CLUSTER 3
SALM
SALB
Log(τ/KA)
EMAX
CLUSTER 2
ICI
MET
Log(τ/KA)
EMAX
CLUSTER 4

Supplementary Figure 14. Graphic representation of pharmacodynamic parameters of $\beta 2_{ADR}$ ligands included in different clusters. Radial graphs were used to represent operational transduction coefficients (Log(τ/KA)) and logistic Emax values. Each radius corresponds to the magnitude of Log(τ/KA) or Emax values. Transduction coefficients are in logarithmic scale, Emax values were normalized to maximal ISO response, and are presented on linear scale. The key specified only for ISO applies to all radial graphs and shows the order in which information for each biosensor is provided.

Supplementary Table 1. Operational and logistic parameters driving ligand segregation into clusters for hMOR responses

Pathway	Cluster A	Cluster B	Cluster C
GαoA		Log(τ)<br>Emax	
Gαi1		Log(τ)<br>Emax	
Gαi2		Log(τ)<br>Emax	
cAMP			Emax
Kir		Emax	Log(τ)
βarr 1	Logτ<br>Emax<br>Log(τ/KA)		
βarr 2	Logτ<br>Emax<br>Log(τ/KA)		
βarr 2-GRK2	Emax	Log(τ)	
βarr 2-GRK5	Log(τ)<br>Emax		
βarr 2-GRK6	Log(τ)<br>Emax		Log(τ/KA)
