# Supplementary material for: Exploring use of unsupervised clustering to associate signaling profiles of GPCR ligands to clinical response
Source: Nat Commun. 2019 Sep 9;10:4075. doi: 10.1038/s41467-019-11875-6 (PMC6733853; doi:10.1038/s41467-019-11875-6)
Supplement: Supplementary file 2 — Description of Additional Supplementary Files [file 41467_2019_11875_MOESM2_ESM.pdf]

Description of Additional Supplementary Files

Supplementary Data 1. Operational and logistic parameters obtained monitoring hMOR responses with BRET-based biosensors

Footnote:
NR: no response
NF: no fitting
MV : missing value : Fits were ambiguous
FA : Full agonist
° Min asymptote fixed to 0
Δ When there was no inflection point within the range of experimental values the Max asymptote in the logistic model was fixed to highest experimental value observed*
* Details of curve fitting in materials and methods

Supplementary Data 2. r2 and p values describing the correlation between frequency of report of undesired events for prescription opioids (standardized gamma scores) and Euclidian distances separating these ligands in the hMOR functional matrix.
Footnote :
Significant correlations, and correlations that explain more than 60% of the variance are highlighted.

$R^2 \geq 0.60$ and $p < 0.05$
$R^2 \geq 0.60$

SD gamma scores for loperamide were only included in correlations for gastrointestinal reports.
* Correlation driven by TRAM, which is also active at NE AND 5-HT transporters. When TRAM was removed correlation was not maintained.

Supplementary Data 3. **Ligand * ligand matrix corresponding to Tanimoto similarity values for opioid ligands derived using Extended-Connectivity Fingerprints (ECFP-6).**
Footnote:
Each value in the matrix is Si,j { Si,j similarity value between compound i and compound j 1= identity 0= no similarity.

Supplementary Data 4. **Ligand * ligand matrix corresponding to Tanimoto similarity values for opioid ligands derived using Functional-Class Fingerprints (FCFP-6).**
Footnote:
Similar considerations as for Supplementary Data 3.

Supplementary Data 5. **Ligand * ligand matrix corresponding to Tanimoto similarity values for opioid ligands derived using MDL MACCS keys (MDL keys).**
Footnote:
Similar considerations as for Supplementary Data 3.

Supplementary Data 6. **r2 and p values describing the correlation between frequency of report of undesired events for prescription opioids (standardized gamma scores) and Euclidian distances separating these ligands in the structural similarity matrix.**
Footnote:

Significant correlations, and correlations that explain more than 60% of the variance are highlighted.

$R^2 \geq 0.60$ and $p < 0.05$
$R^2 \geq 0.60$

Supplementary Data 7. **Operational and logistic parameters obtained monitoring rMOR responses with BRET-based biosensors.**
Footnote:
Considerations for curve fitting as in Supplementary Data 1.

Supplementary Data 8. **Operational and logistic parameters obtained monitoring hDOR responses with BRET-based biosensors.**
Footnote:
Considerations for curve fitting as in Supplementary Data 1.

Supplementary Data 9. **Operational and logistic parameters obtained monitoring rDOR responses with BRET-based biosensors**
Footnote:
Considerations for curve fitting as in Supplementary Data 1.

Supplementary Data 10. **Operational and logistic parameters obtained monitoring BRET and cellular responses elicited by β2ADRs.**
Footnote:
Considerations for curve fitting as in Supplementary Data 1.
*Analysis based on *Mol Pharmacol* **85**, 492-509 (2014).

Supplementary Data 11. **r2 and p values describing the correlation between frequency of report of undesired cardiovascular and respiratory events for prescription β2ADR ligands (standardized gamma scores) and Euclidian distances separating these ligands in the functional matrix.**
Footnote:
Significant correlations are highlighted.

$R^2 \geq 0.60$ and $p < 0.05$
$p < 0.05$

Supplementary Data 12. **Fitted parameter values for the logistic equation and the operational model.** (Compound number are X.Y.Z. X is the profile number. Y is a variation number that can be neglected. And Z is added to compo
