# Supplementary material for: Exploring use of unsupervised clustering to associate signaling profiles of GPCR ligands to clinical response
Source: Nat Commun. 2019 Sep 9;10:4075. doi: 10.1038/s41467-019-11875-6 (PMC6733853; doi:10.1038/s41467-019-11875-6)
Supplement: Supplementary file 15 — Reporting Summary [file 41467_2019_11875_MOESM15_ESM.pdf]

nature research

Corresponding author(s): Graciela Pineyro, Michel Bouvier

Last updated by author(s): Jul 29, 2019

Reporting Summary

Nature Research wishes to improve the reproducibility of the work that we publish. This form provides structure for consistency and transparency in reporting. For further information on Nature Research policies, see Authors & Referees and the Editorial Policy Checklist.

Statistics

For all statistical analyses, confirm that the following items are present in the figure legend, table legend, main text, or Methods section.

n/a	Confirmed	
☐	☒	The exact sample size (*n*) for each experimental group/condition, given as a discrete number and unit of measurement
☐	☒	A statement on whether measurements were taken from distinct samples or whether the same sample was measured repeatedly
☐	☒	The statistical test(s) used AND whether they are one- or two-sided<br>*Only common tests should be described solely by name; describe more complex techniques in the Methods section.*
☐	☒	A description of all covariates tested
☒	☐	A description of any assumptions or corrections, such as tests of normality and adjustment for multiple comparisons
☐	☒	A full description of the statistical parameters including central tendency (e.g. means) or other basic estimates (e.g. regression coefficient) AND variation (e.g. standard deviation) or associated estimates of uncertainty (e.g. confidence intervals)
☒	☐	For null hypothesis testing, the test statistic (e.g. *F*, *t*, *r*) with confidence intervals, effect sizes, degrees of freedom and *P* value noted<br>*Give P values as exact values whenever suitable.*
☒	☐	For Bayesian analysis, information on the choice of priors and Markov chain Monte Carlo settings
☒	☐	For hierarchical and complex designs, identification of the appropriate level for tests and full reporting of outcomes
☒	☐	Estimates of effect sizes (e.g. Cohen's *d*, Pearson's *r*), indicating how they were calculated

Our web collection on statistics for biologists contains articles on many of the points above.

Software and code

Policy information about availability of computer code

Data collection	Adverse event reporting system (AERS)- FDA
Data analysis	Software used for linear correlations, curve fitting and extraction of pharmacodynamic parameters: Graphpad 6; Excel.<br>All clustering and cluster comparisons were conducted using Python 2.7.6. Complete source code is available for download at http://github.com/JonathanGallion/Benredjem-Gallion

For manuscripts utilizing custom algorithms or software that are central to the research but not yet described in published literature, software must be made available to editors/reviewers. We strongly encourage code deposition in a community repository (e.g. GitHub). See the Nature Research guidelines for submitting code & software for further information.

Data

Policy information about availability of data

All manuscripts must include a data availability statement. This statement should provide the following information, where applicable:

- Accession codes, unique identifiers, or web links for publicly available datasets
- A list of figures that have associated raw data
- A description of any restrictions on data availability

All data generated or analyzed in this study are included in the article and supplementary materials or provided as source data files.

Field-specific reporting

Please select the one below that is the best fit for your research. If you are not sure, read the appropriate sections before making your selection.

☒ Life sciences ☐ Behavioural & social sciences ☐ Ecological, evolutionary & environmental sciences

For a reference copy of the document with all sections, see nature.com/documents/nr-reporting-summary-flat.pdf

Life sciences study design

All studies must disclose on these points even when the disclosure is negative.

Sample size	Sample size used was according to standards used in drug screening ranging from n=3-7, except internal standards (Met-ENK), that were ran in all experiments
Data exclusions	Data were excluded only upon evidence of technical difficulties: i.e.: lack of biosensor transfection as indicated by lack of luminescence or total fluorescence readings.
Replication	All BRET biosensors used in the study had been validated in previous publications so readouts were inherently reproducible. All data included in the study was reproducible within the n tested.
Randomization	25 different ligands were tested in human and rat MOR and 21 ligands in human and rat DOR using 10 different BRET readouts. Experiments were carried out in two different laboratories. Drugs, receptors and biosenors were randomly tested between the two labs in a ratio of 1:3 (Bouvier:Pineyro Labs). Guinea pig ileum assays were all run at Pfizer Inc.
Blinding	Experiments were run manually and experimenters who run the experiments also analyzed their own data. There was no blind fold for curve fitting but fitting criteria were fixed a priori guided curve fitting throughout the study.

Behavioural & social sciences study design

All studies must disclose on these points even when the disclosure is negative.

Study description	This study did not include a behavioural & social sciences study design
Research sample	This study did not include a behavioural & social sciences study design
Sampling strategy	This study did not include a behavioural & social sciences study design
Data collection	This study did not include a behavioural & social sciences study design
Timing	This study did not include a behavioural & social sciences study design
Data exclusions	This study did not include a behavioural & social sciences study design
Non-participation	This study did not include a behavioural & social sciences study design
Randomization	This study did not include a behavioural & social sciences study design

Ecological, evolutionary & environmental sciences study design

All studies must disclose on these points even when the disclosure is negative.

Study description	This study did not include an ecological, evoluϴonary & environmental sciences study design
Research sample	This study did not include an ecological, evoluϴonary & environmental sciences study design
Sampling strategy	This study did not include an ecological, evoluϴonary & environmental sciences study design
Data collection	This study did not include an ecological, evoluϴonary & environmental sciences study design
Timing and spatial scale	This study did not include an ecological, evoluϴonary & environmental sciences study design
Data exclusions	This study did not include an ecological, evoluϴonary & environmental sciences study design

Reproducibility	This study did not include an ecological, evoluΘonary & environmental sciences study design
Randomization	This study did not include an ecological, evoluΘonary & environmental sciences study design
Blinding	This study did not include an ecological, evoluΘonary & environmental sciences study design

Did the study involve field work? ☐ Yes ☒ No

Field work, collection and transport

Field conditions	*Describe the study conditions for field work, providing relevant parameters (e.g. temperature, rainfall).*
Location	*State the location of the sampling or experiment, providing relevant parameters (e.g. latitude and longitude, elevation, water depth).*
Access and import/export	*Describe the efforts you have made to access habitats and to collect and import/export your samples in a responsible manner and in compliance with local, national and international laws, noting any permits that were obtained (give the name of the issuing authority, the date of issue, and any identifying information).*
Disturbance	*Describe any disturbance caused by the study and how it was minimized.*

Reporting for specific materials, systems and methods

We require information from authors about some types of materials, experimental systems and methods used in many studies. Here, indicate whether each material, system or method listed is relevant to your study. If you are not sure if a list item applies to your research, read the appropriate section before selecting a response.

Materials & experimental systems

n/a	Involved in the study	
☒	☐	Antibodies
☐	☒	Eukaryotic cell lines
☒	☐	Palaeontology
☐	☒	Animals and other organisms
☒	☐	Human research participants
☒	☐	Clinical data

Methods

n/a	Involved in the study	
☒	☐	ChIP-seq
☒	☐	Flow cytometry
☒	☐	MRI-based neuroimaging

Eukaryotic cell lines

Policy information about cell lines

Cell line source(s)	HEK 293 cells were a kind gift from Dr. Laporte's lab (Methods 92, 19-35 (2016))
Authentication	Cells lines were purchased from ATCC.
Mycoplasma contamination	All cell lines tested negative for mycoplasma contamination.
Commonly misidentified lines (See ICLAC register)	No Commonly misidentified lines were used

Animals and other organisms

Policy information about studies involving animals; ARRIVE guidelines recommended for reporting animal research

Laboratory animals	In vitro assays on guinea pig ileum contratctility were run in the study. Male Hartley guinea pigs were used.
Wild animals	This study did not involve wild animals
Field-collected samples	This study did not involve samples collected from the field
Ethics oversight	All procedures performed on these animals were in accordance with regulations and established guidelines and were reviewed and approved by Pfizer Institutional Animal Care and Use Committee.

Note that full information on the approval of the study protocol must also be provided in the manuscript.
